# Correction: investigating the association between early years foundation stage profile scores and subsequent diagnosis of an autism spectrum disorder: a retrospective study of linked healthcare and education data

**DOI:** 10.1136/bmjpo-2019-000483corr1

**Published:** 2020-03-23

Wright B, Mon-Williams M, Kelly B, *et al*. Investigating the association between early years foundation stage profile scores and subsequent diagnosis of an autism spectrum disorder: a retrospective study of linked healthcare and education data. *BMJ Paediatrics Open* 2019;3:e000483. doi: 10.1136/bmjpo-2019-000483

The license type of the paper has changed from CC BY-NC to CC BY.

© Author(s) (or their employer(s)) 2019. Re-use permitted under CC BY. Published by BMJ.

This is an open access article distributed in accordance with the Creative Commons Attribution 4.0 Unported (CC BY 4.0) license, which permits others to copy, redistribute, remix, transform and build on this work for any purpose, provided the original work is properly cited, a link to the licence is given, and indication of whether changes were made. See: http://creativecommons.org/licenses/by/4.0

