# Supplementary material for: Investigating the association between early years foundation stage profile scores and subsequent diagnosis of an autism spectrum disorder: a retrospective study of linked healthcare and education data
Source: BMJ Paediatr Open. 2019 Nov 11;3(1):e000483. doi: 10.1136/bmjpo-2019-000483 (PMC6863697; doi:10.1136/bmjpo-2019-000483)
Supplement: Supplementary data [file bmjpo-2019-000483supp001.pdf]

Supplementary material 1: Results of logistic regression models

Logistic Regression Models	EYFSP Total Score			EYFSP 5 Item Sub-Score		
	Odds Ratio	95% Confidence Intervals Low	High	Odds Ratio	95% Confidence Intervals Low	High
**Univariate: EYFP score**						
Not low EYSPF score (reference)						
Low EYSFP score	23.7*	12.9	43.8	39.9*	21.6	73.8
**Multivariate: EYFP score and covariates**						
Not low EYSPF score (reference)						
Low EYSFP score	29.4*	14.4	59.8	58.1*	27.4	123.0
Gender: Female (reference)						
Gender: Male	2.87*	1.56	5.29	2.38*	1.28	4.41
Child ethnicity: White British (reference)						
Pakistani Heritage	0.45*	0.27	0.76	0.42*	0.24	0.71
Other	0.69	0.33	1.41	0.63	0.30	1.32
Receiving free school meals (reference)						
Not receiving free school meals	2.29*	1.25	4.19	2.39*	1.30	4.41
Age (years) at GP data extract: Five (reference)						
Six	1.60	0.54	4.76	1.16	0.38	3.54
Seven	0.94	0.31	2.80	0.67	0.22	2.05
Eight plus	0.93	0.31	2.82	0.71	0.23	2.19

* = significant at the 0.05 level

Wright B, *et al. BMJ Paediatrics Open* 2020; 3:e000483. doi: 10.1136/bmjpo-2019-000483

Supplementary material 2: Read codes for autism

We used the Read codes below to identify children with a diagnosis of autism. This was supplemented by a free text search (which did not identify further cases).

**Read code**	**Read code description**
**ASDs**	
E140.00	Infantile autism
E140000	Active infantile autism
E140100	Residual infantile autism
E140.12	Autism**
E140.13	Childhood autism
E140000	Active infantile autism
E140z00	Infantile autism NOS
Eu84000	[X]Childhood autism
Eu84011	[X]Autistic disorder**
Eu84012	[X]Infantile autism
Eu84100	[X]Atypical autism
Eu84z11	[X]Autistic spectrum disorder
Eu84500	[X]Asperger's syndrome**

Wright B, *et al. BMJ Paediatrics Open* 2020; 3:e000483. doi: 10.1136/bmjpo-2019-000483

Supplementary material 3: The distribution of EYFSP scores for children with and without a diagnosis of autism spectrum disorder

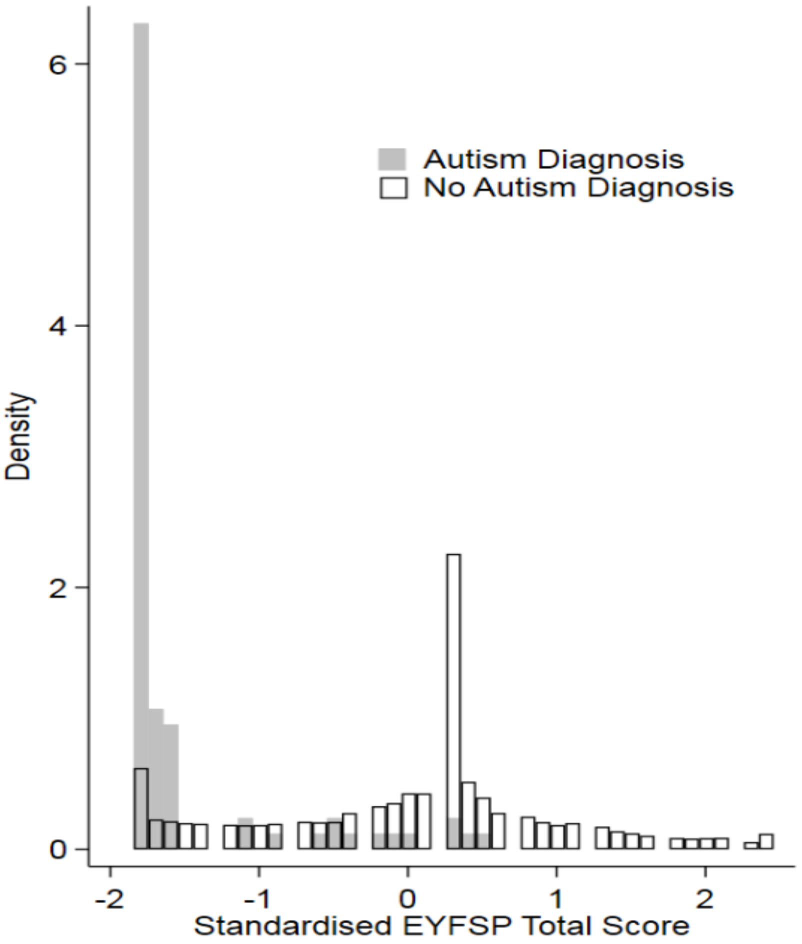

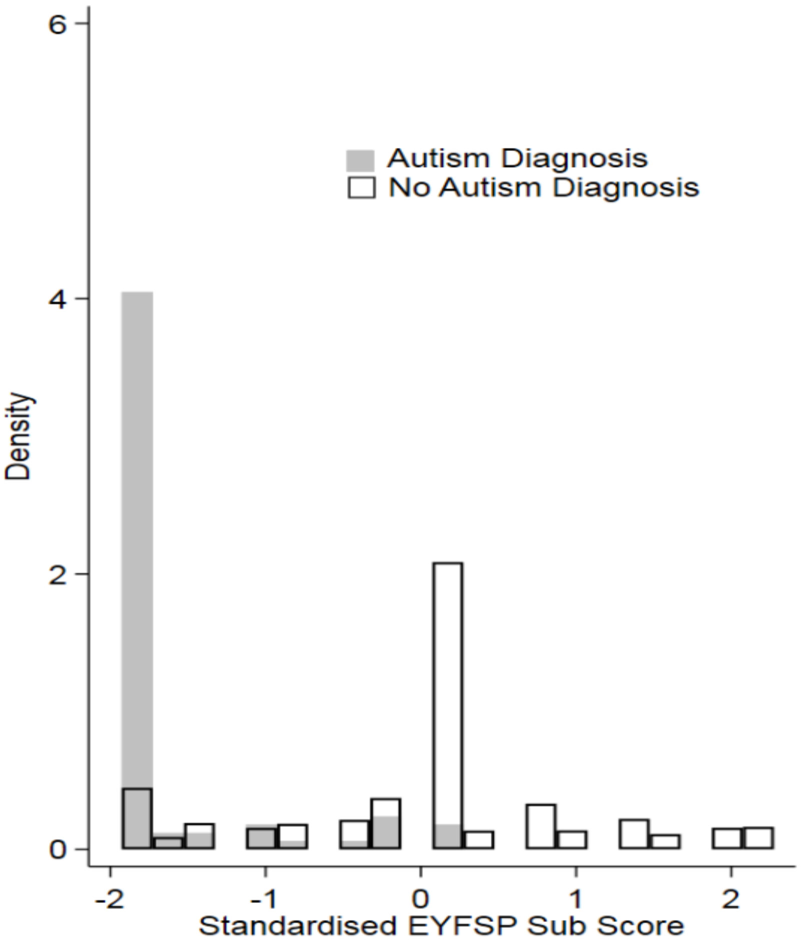

Wright B, *et al. BMJ Paediatrics Open* 2020; 3:e000483. doi: 10.1136/bmjpo-2019-000483
