# Supplementary material for: Investigating the association between early years foundation stage profile scores and subsequent diagnosis of an autism spectrum disorder: a retrospective study of linked healthcare and education data
Source: BMJ Paediatr Open. 2019 Nov 11;3(1):e000483. doi: 10.1136/bmjpo-2019-000483 (PMC6863697; doi:10.1136/bmjpo-2019-000483)

BMJ Paediatrics Open

BMJ Paediatrics Open is committed to open peer review. As part of this commitment we make the peer review history of every article we publish publicly available.

When an article is published we post the peer reviewers' comments and the authors' responses online. We also post the versions of the paper that were used during peer review. These are the versions that the peer review comments apply to.

The versions of the paper that follow are the versions that were submitted during the peer review process. They are not the versions of record or the final published versions. They should not be cited or distributed as the published version of this manuscript.

BMJ Paediatrics Open is an open access journal and the full, final, typeset and author-corrected version of record of the manuscript is available on our site with no access controls, subscription charges or pay-per-view fees (http://bmjpaedsopen.bmj.com).

If you have any questions on BMJ Paediatrics Open's open peer review process please email info.bmjpo@bmj.com

BMJ Paediatrics Open

Investigating the association between early years foundation stage profile (EYFSP) scores and subsequent diagnosis of an autism spectrum disorder: a retrospective study of linked healthcare and education data

Journal:	*BMJ Paediatrics Open*
Manuscript ID	bmjpo-2019-000483
Article Type:	Original article
Date Submitted by the Author:	14-Mar-2019

Complete List of Authors:	Wright, Barry; University of York, Health Sciences<br>Mon-Williams, Mark; University of Leeds, Institute of Psychological Sciences<br>Kelly, Brian; Bradford Institute for Health Research<br>Williams, Stefan; Bradford Teaching Hospitals NHS Foundation Trust<br>Sims, David; Bradford District Care NHS Foundation Trust<br>Mushtaq, Faisal; University of Leeds, Institute of Psychological Sciences<br>Wright, Prof John; Bradford Teaching Hospitals NHS Foundatin Trust, Bradford Institute for Health Research

Keywords:	Autism, Screening, Data Collection, Health Service

SCHOLARONE™
Manuscripts

Investigating the association between early years foundation stage profile (EYFSP) scores and subsequent diagnosis of an autism spectrum disorder: a retrospective study of linked healthcare and education data

Barry Wright[1], Mark Mon-Williams[2,3], Brian Kelly[3], Stefan Williams[4], David Sims[5], Faisal Mushtaq[2], John Wright[3]

Affiliations:

1. Hull York Medical School, University of York, UK

2. School of Psychology, University of Leeds, UK

3. Bradford Institute for Health Research, Bradford, UK

4. Bradford Hospitals NHS Teaching Hospitals NHS Foundation Trust

5. Bradford District Care NHS Foundation Trust

Corresponding author

Professor Barry Wright

Hull York Medical School

University of York

York

YO10 5DD

UK

Email: Barry.wright1@nhs.net

Telephone number: 01904 724540

Word count: 2420

nfidential: For Review Only

Abstract

Objective: We set out to test whether the Early Years Foundation Stage Profile score derived from 17 items assessed by teachers at the end of reception school year had any association with Autism Spectrum Disorders diagnosis in subsequent years. This study tested the feasibility of successfully linking education and health data.

Design: A retrospective data linkage study.

Setting and participants: The Born in Bradford longitudinal cohort of 13,500 children.

Outcome measures: We linked the 'total' score of the Early Years Foundation Stage Profile (EYFSP) score at the end of reception year with subsequent diagnosis of an autism spectrum disorder, using all ASD general practitioner Read codes. We prospectively identified 5 key items in the EYFSP using a panel of early years autism experts and also tested the association of this 'weighted' score.

Results: This study demonstrated the feasibility of linking education and health data using Autism Spectrum Disorders as an exemplar. The rate of diagnosis of Autism Spectrum Disorders at follow up was 1%, children scoring below 25 on the total EYFSP had a 4.1% chance of ASD compared to 0.15% of the remaining children. Using the prospectively designed sub-score this difference was greater (6.4% and 0.12% respectively).

Conclusions: It is feasible to link education and health data. Performance on teacher ratings taken universally in school reception class can flag children at risk of autism spectrum disorders. Further research is warranted to explore the usefulness of EYFSP as an initial screening tool for ASD in early school years.

Confidential: For Review O

What is already known about this topic?

- Routine education and health data is rarely linked.
- To date there is limited evidence that screening for autism spectrum disorders in the first 6 years of life is cost-effective.

What this study adds?

- It is feasible to link routine education and health data.
- Performance on educational measures taken universally in school reception class can flag children at risk of autism spectrum disorders.
- Linking this data has the potential to decrease costs associated with undiagnosed childhood conditions to the individual as well as health and education services.

This is an Open Access article distributed in accordance with the Creative Commons Attribution Non Commercial Licence (CC BY NC – 4.0) which permits others to distribute and build on this work non-commercially, provided the original work is properly cited and the use is non-commercial.

Introduction

Large quantities of electronic data are generated routinely in health and education sectors. Linking these data could present an opportunity to improve information sharing and facilitate service user transitions within and across systems.

10% of children and young people aged 5–16 years have a clinically diagnosed mental disorder [1]. The short-term costs are estimated to be £1.58 billion per annum, with annual long-term costs calculated at £2.35 billion [2]. Neurodevelopmental impairments and conditions with high levels of need contributing to this cost have an estimated prevalence of around 3–4% of children in England [2]. The CMO suggested that "*Commissioners and providers of services to children*

in primary education should develop and agree arrangements to ensure all primary schools adopt a comprehensive whole school approach to children's social and emotional wellbeing. They should provide specific help for those children most at risk (or already showing signs) of social, emotional and behavioural problems" [2]. The challenge facing policy makers and providers is how such a suggestion might be implemented. A useful starting point might arise from linking routine data across health and education services. ASD presents an exemplar condition that could elucidate the challenges and potential utility of data linkage across domains.

Autism Spectrum Disorders are neurodevelopmental disorders that lead to impaired reciprocal social interaction and repetitive or restricted patterns of behaviour [3] occurring in at least 1% of children in the UK [4]. The behavioural problems associated with the condition are a major cause of children being excluded from school [2]. A recent review showed that government policies and community resources impact upon early identification of ASD [5] with evidence of geographic variation. Socio-economic status and level of parental concern affect age of diagnosis [5]. Parents experience high levels of stress with the ASD diagnostic process, with over half dissatisfied with current UK services [6]. On average, families have to wait 3-4 years to receive a diagnosis [[6, 7]. Many children are identified early with a range of difficulties but not given a diagnosis of autism until much later [7]. Box 1 provides typical testimony from a parent consulted in our patient and public involvement work (slightly abridged).

Box 1: *We requested a referral to paediatrician as we suspected autism when my son was four years. The SENCO in his nursery had been involved when he was 3.5 years old as staff said he wasn't listening and his attention span was poor. We approached our health visitor after this to express our concerns. They referred him for speech and language therapy. The*

speech and language therapist referred him to the Paediatrician. We waited nearly 2 years to be seen. After an initial appointment we were told they wanted to wait and see. This meant that our son did not get appropriate treatment until he was eight years of age. Should there not be a more systematic way of assessing children in need early?

Early intervention in ASD is associated with long-term symptom reduction [8]. This includes identifying appropriate educational placement early and parenting support interventions [8]. There has been a call for more sophisticated approaches to screening (such as stepped approaches or at risk group identification) since whole population approaches have not proved cost-effective [9].

A universal educational assessment is conducted on *all* children in their first year of schooling in the UK (the Early Years Foundation Stage Profile; EYFSP).We predicted that scores on this developmental assessment might identify children at risk of neurodevelopmental problems. This could lead to earlier intervention to prevent the poor outcomes associated with undiagnosed conditions. Our prediction was motivated by the fact that information collected through the EYFSP routinely covers all the domains of autism symptomatology, providing information about child's language development, social skills and emotional development.

Methods

Design

This was a retrospective data linkage study. To test our hypothesis, we focused on ASD as an exemplar and retrospectively examined the EYFSP scores collected on the 13,500 children within the Born in Bradford longitudinal birth cohort study [10]. This cohort has been followed

up since birth. The BiB cohort comprises 12,453 women recruited at 28 weeks of pregnancy, who gave birth at the Bradford Royal Infirmary to 13,857 children between the period 2007 and 2011 [10]. Half of all BiB families live within wards classed amongst the 20% most deprived within England and Wales, and 45% are of Pakistani origin [10].

Patient and public involvement

A key strength of BiB has been its effective community engagement with considerable time and effort invested to ensure genuine co-production of research. There is an active parent governor group and they advised on the protocol and materials used for this study. BiB has an active approach to partnership with families through regular newsletters, Facebook, and Twitter with regular family network days and an annual family science festival.

Full informed consent was obtained for all participants. Ethical approval was granted by Bradford NHS Research Ethics Committee (Ref 07/H1302/112). Cohort members gave their consent to access GP records via SystmOne, which currently has complete coverage of all GP practices in Bradford. Figure 1 provides an overview of the recruitment process and the final sample included in the current study. Table 1 provides a comparison of the characteristics of the current sample with the whole Born in Bradford cohort.

Confidential: For Review Only

Table 1. Sample characteristics

	Sample in the current study (n = 8,935)	Sample in the whole cohort (n = 13857)
**Gender**		
Female	49.1%	49.0%
Male	50.9%	51.0%
**Ethnicity**		
White British	36.1%	37.0%
Pakistani Heritage	51.8%	48.6%
Other	12.1%	14.4%
**Free school meal status**		
In receipt	24.7%	25.1%
Not in Receipt	75.3%	74.9%

Outcome measures

Early years foundation stage profile score

Table 2 displays the 17 items of The Early Years Foundation Stage Profile, each scored 2 (meeting the level of development *expected*), 3 (*exceeding* this level) or 1 (not yet reaching this level - *emerging*). The items are designed to measure a range of educational, socio-emotional, communicative and developmental factors. We used the total EYFSP score (the 'total score') and a sub-score developed by a small group of ASD assessment experts prior to study and blind

Confidential: For Review Only

to the results (a 5 item ‘sub-score’) (see table 3). The experts were academic and clinical child psychiatrists and psychologists with many years of ASD experience between them. The five items were chosen from the four main symptom areas defined in the World Health Organisation (1992) [3] research diagnostic criteria for ASD namely social reciprocity, language and communication, imagination delays and repetitive and stereotyped patterns of behaviour. Because the social reciprocity domain is given more weight in this classification system it was decided to include two items from the EYFSP to reflect this weighting.

Sensitivity analysis was carried out using the scores derived from all items.

Read Code Diagnosis of ASD

The outcome measure for this study was the presence of a ‘Read code’ for an ASD recorded in a child’s primary care records. A validated Read code list (see supplementary material 3) has been shown in previous published work in ASD to be reliable and used with confidence to study ASD [11].

Confidential: For Review Only

Table 2 : Early Years Skills Foundation Profile: All items

Physical development: Health and self-care	EYFSP05
Communication and language: Listening and attention	EYFSP01
Personal, social and emotional: Managing feelings and behaviour	EYFSP07
Communication and language: Understanding	EYFSP02
Expressive arts and design: Being imaginative	EYFSP17
Understanding the world: People and communities	EYFSP13
Personal, social and emotional: Self-confidence and self-awareness	EYFSP06
Communication and language: Speaking	EYFSP03
Personal, social and emotional: Making relationships	EYFSP08
Expressive arts and design: Exploring and using media and materials	EYFSP16
Mathematics: Shapes, space and measures	EYFSP12
Physical development: Moving and handling	EYFSP04
Understanding the world: The world	EYFSP14
Mathematics: Numbers	EYFSP11
Literacy: Reading	EYFSP09
Literacy: Writing	EYFSP10
Understanding the world: Technology	EYFSP15

Confidential: For Review Only

Table 3: Early Years Skills Foundation Profile (EYFSP): Weighted sub-score
Using a weighted sub-score, where four aspects of childhood autism (social, language and communications, imagination and repetitive behaviour) were mapped onto EYFSP elements
**The social aspect mapped onto:**
• Personal, social and emotional: Managing feelings and behaviour/
• Personal, social and emotional: Making relationships
**Language and communications aspect mapped onto:**
• Communication and language: Listening and attention
**Imagination aspect mapped onto:**
• Expressive arts and design: Being imaginative
**Repetitive and stereotyped behaviours mapped onto:**
• Physical development: Health and self-care

Confidential: For Review Only

Procedure

Linkage was carried out using NHS number, surname and date of birth (99% of children matched) and education records. At the point of analysis, EYFSP results were available for 9,029 children, with 8,854 of these children matched to GP records (98%).

Analysis

Logistic regression was employed to model the relationship between EYFSP score and the outcome of ASD diagnosis using Stata 13 [12]. A number of covariates were included in the analysis; gender (classified as male or female), ethnicity (White British, Pakistani Heritage and Other), free school meal status (whether in receipt of free school meals or not) and the age of the child (in years) at the date of GP data extract. Marginal effects were estimated [13] to produce predicted rates of ASD diagnosis for children based on whether they had a low EYFSP score.

The total EYFSP score ranged from 17 to 51, with a mean of 31.8 and a standard deviation of 8.0. The five item sub-score ranged from 7 to 21, with a mean of 13.6 and a standard deviation of 3.4 (see figure 2). However, the EYFSP scores are not normally distributed; mostly children score average on each item, but there are some who score higher and a fairly distinct group of children who have low scores resulting in a bimodal distribution of EYFSP scores, as indicated in figure 3. For this reason the EYFSP scores are dichotomised for the purposes of analysis. Those scoring below 25 in the total EYFSP score and below 10 in the five item sub-score were categorised as low scores; these cut points equate to scores less than one standard deviation below the mean (see figure 3).

Confidential: For Review Only

Results

We found that children with a low EYFSP sub-score were approximately 50 times more likely to have a diagnosis of autism compared to children without a low score. Males were twice as likely to have a diagnosis of ASD, whilst White British children and children not in receipt of free school meals had higher rates of diagnosis recorded in the GP records, apart from age of the child, all differences were statistically significant and all covariates improved the model (see supplementary material 1 for full model results). Sensitivity analysis was carried out using the scores derived from all items and produced similar results; though the association was stronger for the EYFSP sub-scores (see Figure 4).

Confidential: For Review Only

Children with a low total EYFSP score (A) have a rate of autism of around 41 per 1,000 children (4.10%, 95% CI: 3.08 - 5.13), far higher than children who do not have a low total EYFSP score (0.15%, 95% CI: 0.05 - 0.25).

Children with a low five item EYFSP sub-score (B) have a rate of autism of around 64 per 1,000 children (6.38%, 95% CI: 4.76 - 8.00), far higher than children who do not have a low five item EYFSP sub-score (0.12%, 95% CI: 0.04 - 0.20).

Confiden

Discussion

Our linkage of routine education and health data has revealed an association between school EYFSP score profile and the diagnosis of ASD. We focused this proof-of-concept study on ASD as: (i) the issues with late diagnosis are well documented; (ii) early diagnosis is known to lead to enhanced parental interventions and (iii) improved educational pathways lead to better outcomes. The linkage process and subsequent analyses of the linked datasets has illustrated a potentially powerful way to create a smoother, more joined-up and timely early patient pathway to autism assessment by linking data across the currently tortuous pathway to referral for ASD assessment. Gold standard assessment for ASD is lengthy and expensive [14] so care needs to be taken to ensure that screening has good specificity and sensitivity to avoid large numbers of unnecessary assessments. Further research is therefore necessary. Population screening for ASD is also expensive [14] and to date, the effectiveness of different screening strategies for ASD in reducing time to diagnosis has not been adequately tested [9]. Whilst researchers have recommended that screening for ASD only be carried out in 'at risk' groups [15], no localities or nations have yet successfully implemented or researched this. Our study suggests that a promising focus for future research lies in the use of routine educational data to identify children

at risk. This could be highly cost-effective given the universal availability of this educational data.

Clinical and Research Implications

Encouraged by strong stakeholder support, we are now prospectively researching whether children identified by the EYFSP is a suitable 'at risk' group for specific ASD screening and assessment for ASD in terms of acceptability, ethics and cost-effectiveness, and whether it is a sensitive and specific method of enabling prompt assessment leading to early intervention.

If successful it would substantially increase efficiencies in the delivery of current services. These include assessment resources and appropriate school placement/support earlier. It may also prevent multiple assessments from different professionals over time. We have recently shown an inequality of diagnosis as a function of socioeconomic position [16] and the use of routine linked health and education data could be a powerful tool to help tackle such health inequalities.

Routine electronic data that are relevant to health and care pathways exist in fragments, spread across multiple organisations, each fragment with its own data controller. The challenges of cross-organisation coordination and information governance make it difficult to obtain linked data, and so most care providers and researchers have been unable to use it as a means of understanding whole-system healthcare. In Bradford, we have addressed this as part of the Connected Health Cities project, by reaching data sharing agreements with each of the health and social care organisations in the Bradford city region. Identifiable information is removed at source, and so personal information is not available to the project, which enables whole population data to be analysed. The pseudonym is derived from the NHS number, which crosses organisations, so that data can be linked after pseudonymisation. School UPN numbers are input

For Review Only

into the electronic health record by the school nurse service. There is good scope for this approach to be used across multiple Trusts.

Conclusions

Our 'proof-of-concept' study suggests that linking education and health data could improve the detection and support of children with neurodevelopmental problems such as ASD earlier – a priority area identified by the Chief Medical Officer [2]. The use of linked data to benefit outcomes is an important future goal [17]. In this context our demonstration has implications beyond autism. It is becoming clear that the routine linkage of education and health data has the potential to drive efficiencies in children's services, facilitate early intervention and ultimately, improve quality of life for large numbers of children and their families.

Acknowledgments

Acknowledgements: Connected Health Cities is a Northern Health Science Alliance led programme funded by the Department of Health and delivered by a consortium of academic and NHS organisations across the north of England. The work uses data provided by patients and collected by the NHS as part of their care and support. The views expressed are those of the author(s) and not necessarily those of the NHSA, NHS or the Department of Health.

Funding: This research received no specific grant from any funding agency in the public, commercial or not-for-profit sectors.

Competing interests: The authors have declared that no competing interests exist.

Authors contributions: Contributed to the writing of the manuscript: BW, MM-W, BK, SW, DS, FM, JW. Statistical analysis BK, Agree with the manuscript's results and conclusions: BW, MM-W, BK, SW, DS, FM, JW. All authors have read, and confirm that they meet, ICMJE criteria for authorship.

Abbreviations: ASD - Autism Spectrum Disorders; CMO – Chief Medical Officer; EYFSP – Early Years Foundation Stage Profile Score; NHS – National Health Service;

https://mc.manuscriptcentral.com/bmjpo

Provenance: Not commissioned; externally peer-reviewed

Data sharing statement: The Born in Bradford (BiB) dataset is available from: https://borninbradford.nhs.uk/research/how-to-access-data/

Confidential: For Review Only

References

1. NICE Local Government Briefings. Social and emotional wellbeing for children and young people. 25 September 2013.

2. Chief Medical Officer's annual report 2012: Our Children Deserve Better: Prevention Pays (published Oct 2013)

3. World Health Organization. The ICD-10 classification of mental and behavioural disorders: diagnostic criteria for research, 1992.

4. Baird, G., Simonoff, E., Pickles, A., Chandler, S., Loucas, T., Meldrum, D. and Charman, T. Prevalence of disorders of the autism spectrum in a population cohort of children in South Thames: the Special Needs and Autism Project (SNAP). *Lancet* 2006; 368(9531): 210-215.

5. Daniels AM, Mandell D. Explaining differences in age at autism spectrum disorder diagnosis: A critical review. *Autism* 2013; 18(5): 583-597.

6. Crane L, Chester JW, Goddard L, Henry LA, Hill E. Experiences of autism diagnosis: A survey of over 1000 parents in the United Kingdom. Autism 2016; 20(2): 153-162.

7. Care Quality Commission. Health Care for Disabled Children and Young People. 2012.

8. Pickles A, Le Couteur A, Leadbitter K, Salomone E, Cole-Fletcher R, Tobin H, Gammer I, Lowry J, Vamvakas G, Byford S, Aldred C. Parent-mediated social communication therapy for young children with autism (PACT): long-term follow-up of a randomised controlled trial. *The Lancet*, 2016; 388(10059): 2501-2509.

9. Daniels AM, Halladay AK, Shih A, Elder LM, Dawson G. Approaches to enhancing the early detection of autism spectrum disorders: a systematic review of the literature. *Journal of the American Academy of Child & Adolescent Psychiatry,* 2014; 53(2): 141-152.

10. Wright J, Small N, Raynor P, Tuffnell D, Bhopal R, Cameron N, Fairley L, Lawlor DA, Parslow R, Petherick ES.
and Pickett KE.. Cohort Profile: the Born in Bradford multi-ethnic family cohort study. *International Journal of Epidemiology* 2013; 42(4): 978–91.

11. Hagberg, K,W., & Jick, S.S. Validation of autism spectrum disorder diagnoses recorded in the Clinical Practice Research Datalink, 1990–2014. *Clinical Epidemiology*, 2017; *9*, p.475

12. StataCorp. Stata Statistical Software: Release 13. College Station, Texas: StataCorp LP: 2013.

13. Williams R. Using the margins command to estimate and interpret adjusted predictions and marginal effects. *Stata Journal* 2012; 12: 308–331.

Confidential: For Review Only

14. Falkmer T, Anderson K, Falkmer M, Horlin C. Diagnostic procedures in autism spectrum disorders: a systematic literature review. *European child & adolescent psychiatry* 2013; 22(6): 329-340.

15. Towle PO, Patrick PA. Autism Spectrum Disorder Screening Instruments for Very Young Children: A Systematic Review. *Autism Research and Treatment*, 2016.

16. Kelly B, Williams S, Collins S, Mushtaq F, Mon-Williams M, Wright B, Mason D, Wright J. The association between socioeconomic status and autism diagnosis in the United Kingdom for children aged 5–8 years of age: Findings from the Born in Bradford cohort. *Autism* 2017; Nov 7:1362361317733182.

17. Schneeweiss S. Learning from big health care data. *New England Journal of Medicine* 2014; 370(23): 2161-2163.

Figure legends

Figure 1: Recruitment flow chart

Figure 2: Early Years Foundation Stage Profile (EYFSP): Weighted sub-score

Figure 3: Distribution of EYFSP Scores - standardised with standard deviation shown

Figure 4: Predicted rates of ASD for children by (A) total EYFSP score and (B) five item EYFSP sub-score (designed to map more closely to items that measure factors associated with autism)

Confidential: For Review Only

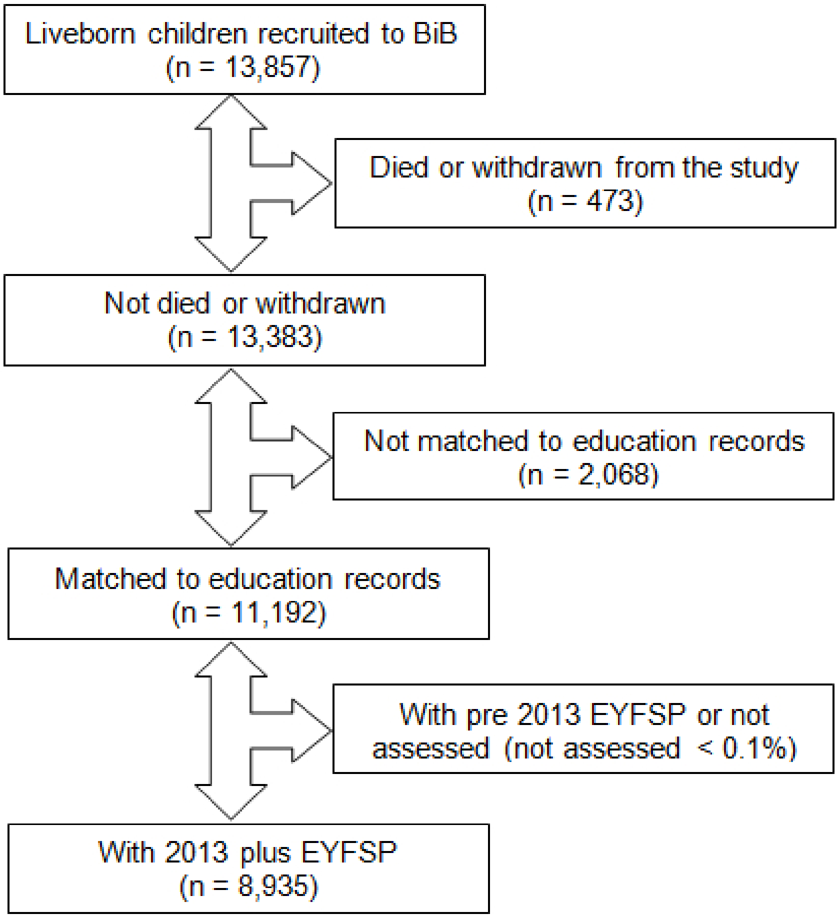
Liveborn children recruited to BiB
(n = 13,857)
Died or withdrawn from the study
(n = 473)
Not died or withdrawn
(n = 13,383)
Not matched to education records
(n = 2,068)
Matched to education records
(n = 11,192)
With pre 2013 EYFSP or not
assessed (not assessed < 0.1%)
With 2013 plus EYFSP
(n = 8,935)

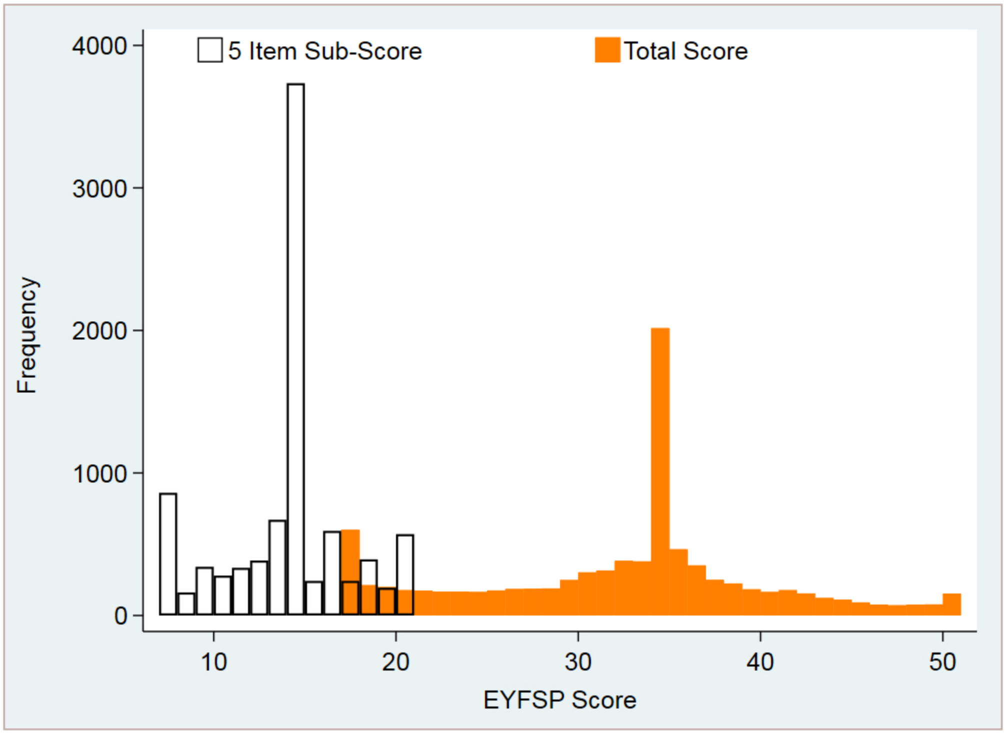

352x256mm (72 x 72 DPI)

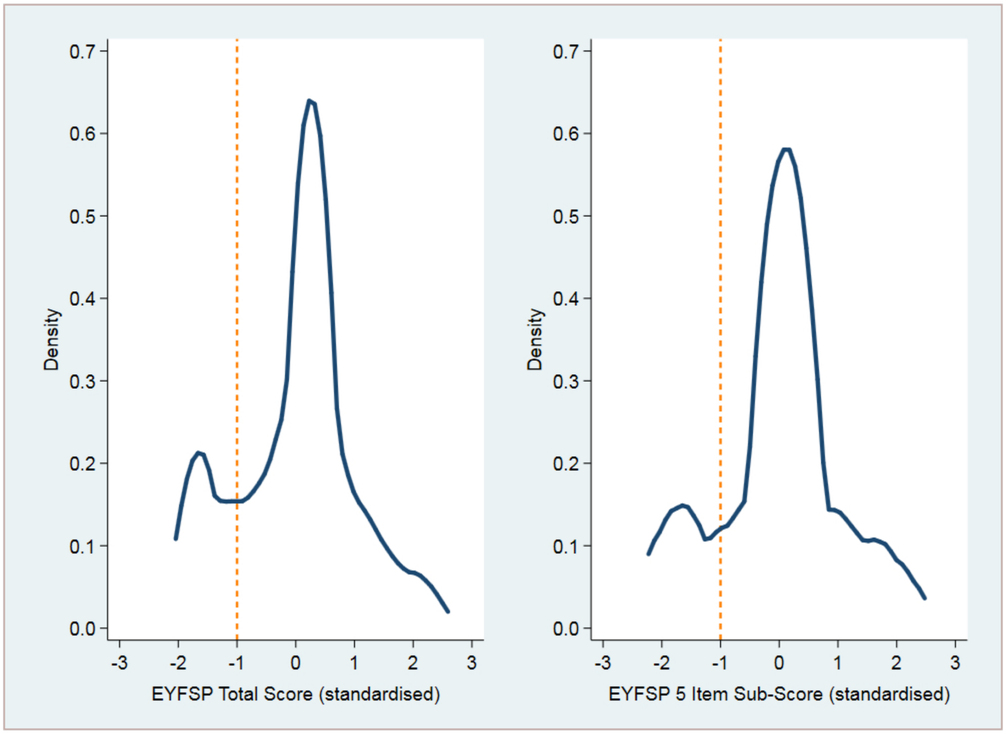

352x256mm (72 x 72 DPI)

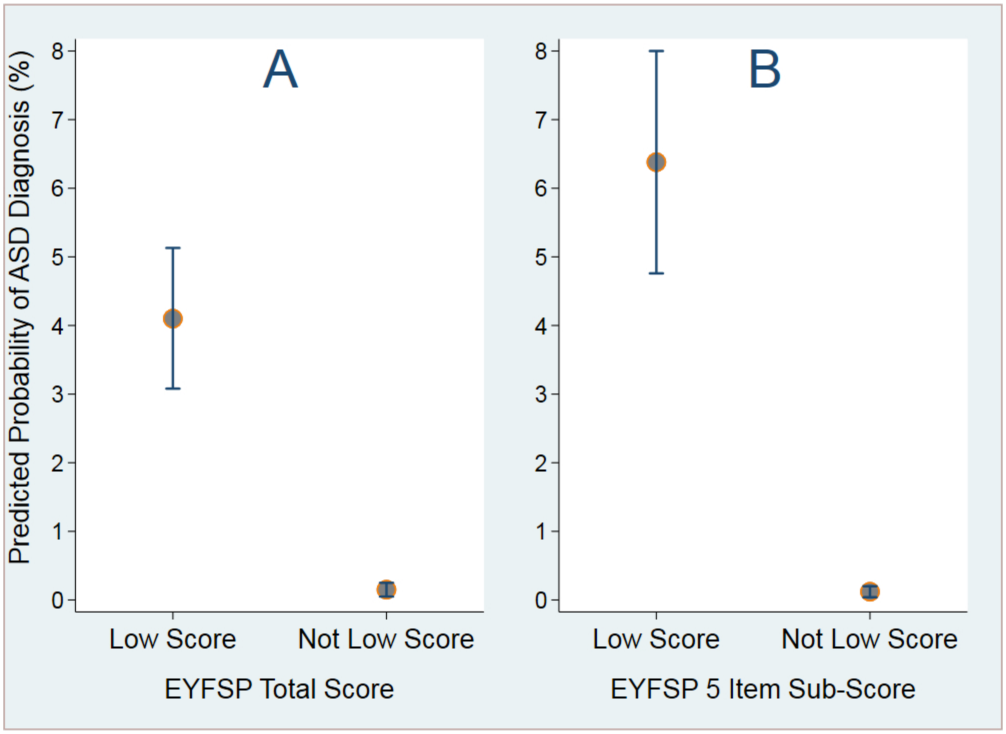

352x256mm (72 x 72 DPI)

Supplementary material 1: Results of logistic regression models

Logistic Regression Models	EYFSP Total Score			EYFSP 5 Item Sub-Score		
	Odds Ratio	95% Confidence Intervals Low	95% Confidence Intervals High	Odds Ratio	95% Confidence Intervals Low	95% Confidence Intervals High
**Univariate: EYFSP score**						
Not low EYFSP score (reference)						
Low EYFSP score	23.7*	12.9	43.8	39.9*	21.6	73.8
**Multivariate: EYFSP score and covariates**						
Not low EYFSP score (reference)						
Low EYFSP score	29.4*	14.4	59.8	58.1*	27.4	123.0
Gender: Female (reference)						
Gender: Male	2.87*	1.56	5.29	2.38*	1.28	4.41
Child ethnicity: White British (reference)						
Pakistani Heritage	0.45*	0.27	0.76	0.42*	0.24	0.71
Other	0.69	0.33	1.41	0.63	0.30	1.32
Receiving free school meals (reference)						
Not receiving free school meals	2.29*	1.25	4.19	2.39*	1.30	4.41

Confidential: For Review Only

Age (years) at GP data extract: Five (reference)						
Six	1.60	0.54	4.76	1.16	0.38	3.54
Seven	0.94	0.31	2.80	0.67	0.22	2.05
Eight plus	0.93	0.31	2.82	0.71	0.23	2.19

* = significant at the 0.05 level

Confidential: For Review Only

Supplementary material 2: Kernel density of the total score and the weighted sub-score

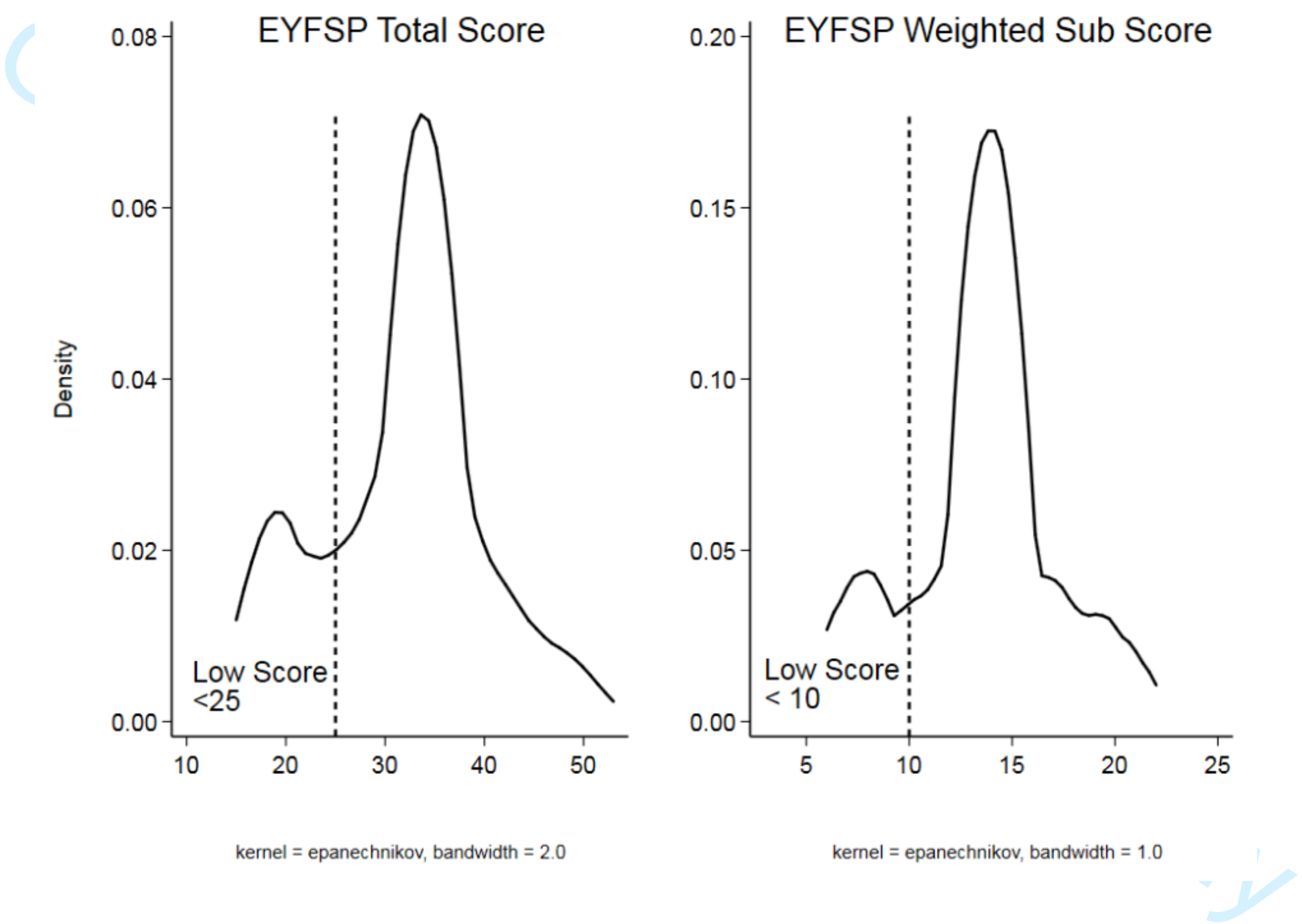

Supplementary material 3: Read codes for autism

We used the Read codes below to identify children with a diagnosis of autism. This was supplemented by a free text search (which did not identify further cases).

**Read code**	**Read code description**
**ASDs**	
E140.00	Infantile autism
E140000	Active infantile autism
E140100	Residual infantile autism
E140.12	Autism**
E140.13	Childhood autism
E140000	Active infantile autism
E140z00	Infantile autism NOS
Eu84000	[X]Childhood autism
Eu84011	[X]Autistic disorder**
Eu84012	[X]Infantile autism
Eu84100	[X]Atypical autism
Eu84z11	[X]Autistic spectrum disorder
Eu84500	[X]Asperger's syndrome**

Confidential: For Review Only

BMJ Paediatrics Open

Investigating the association between early years foundation stage profile (EYFSP) scores and subsequent diagnosis of an autism spectrum disorder: a retrospective study of linked healthcare and education data

Journal:	*BMJ Paediatrics Open*
Manuscript ID	bmjpo-2019-000483.R1
Article Type:	Original article
Date Submitted by the Author:	11-Apr-2019

Complete List of Authors:	Wright, Barry; University of York, Health Sciences<br>Mon-Williams, Mark; University of Leeds, Institute of Psychological Sciences<br>Kelly, Brian; Bradford Institute for Health Research<br>Williams, Stefan; Bradford Teaching Hospitals NHS Foundation Trust<br>Sims, David; Bradford District Care NHS Foundation Trust<br>Mushtaq, Faisal; University of Leeds, Institute of Psychological Sciences<br>Wright, Prof John; Bradford Teaching Hospitals NHS Foundatin Trust, Bradford Institute for Health Research

Keywords:	Autism, Screening, Data Collection, Health Service

SCHOLARONE™
Manuscripts

Investigating the association between early years foundation stage profile (EYFSP) scores and subsequent diagnosis of an autism spectrum disorder: a retrospective study of linked healthcare and education data

Barry Wright[1], Mark Mon-Williams[2,3], Brian Kelly[3], Stefan Williams[4], David Sims[5], Faisal Mushtaq[2], John Wright[3]

Affiliations:

1. Hull York Medical School, University of York, UK

2. School of Psychology, University of Leeds, UK

3. Bradford Institute for Health Research, Bradford, UK

4. Bradford Hospitals NHS Teaching Hospitals NHS Foundation Trust

5. Bradford District Care NHS Foundation Trust

Corresponding author

Professor Barry Wright

Hull York Medical School

University of York

York

YO10 5DD

UK

Email: Barry.wright1@nhs.net

Telephone number: 01904 724540

Confidential: For Review Only

Abstract

Objective: We set out to test whether the Early Years Foundation Stage Profile score derived from 17 items assessed by teachers at the end of reception school year had any association with Autism Spectrum Disorders diagnosis in subsequent years. This study tested the feasibility of successfully linking education and health data.

Design: A retrospective data linkage study.

Setting and participants: The Born in Bradford longitudinal cohort of 13,500 children.

Outcome measures: We linked the 'total' score of the Early Years Foundation Stage Profile (EYFSP) score at the end of reception year with subsequent diagnosis of an autism spectrum disorder, using all ASD general practitioner Read codes. We prospectively identified 5 key items in the EYFSP using a panel of early years autism experts and also tested the association of this 'weighted' score.

Results: This study demonstrated the feasibility of linking education and health data using Autism Spectrum Disorders as an exemplar. The rate of diagnosis of Autism Spectrum Disorders at follow up was 1%, children scoring below 25 on the total EYFSP had a 4.1% chance of ASD compared to 0.15% of the remaining children. Using the prospectively designed sub-score this difference was greater (6.4% and 0.12% respectively).

Conclusions: It is feasible to link education and health data. Performance on teacher ratings taken universally in school reception class can flag children at risk of autism spectrum disorders. Further research is warranted to explore the usefulness of EYFSP as an initial screening tool for ASD in early school years.

What is already known about this topic?

- Routine education and health data is rarely linked.
- To date there is limited evidence that screening for autism spectrum disorders in the first 6 years of life is cost-effective.

Confidential: For Review O

What this study adds?

- It is feasible to link routine education and health data.
- Performance on educational measures taken universally in school reception class can flag children at risk of autism spectrum disorders.
- Linking this data has the potential to decrease costs associated with undiagnosed childhood conditions to the individual as well as health and education services.

This is an Open Access article distributed in accordance with the Creative Commons Attribution Non Commercial Licence (CC BY NC – 4.0) which permits others to distribute and build on this work non-commercially, provided the original work is properly cited and the use is non-commercial.

Introduction

Large quantities of electronic data are generated routinely in health and education sectors. Linking these data could present an opportunity to improve information sharing and facilitate service user transitions within and across systems.

10% of children and young people aged 5–16 years have a clinically diagnosed mental disorder [1]. The short-term costs are estimated to be £1.58 billion per annum, with annual long-term costs calculated at £2.35 billion [2]. Neurodevelopmental impairments and conditions with high levels of need contributing to this cost have an estimated prevalence of around 3–4% of children in England [2]. The CMO suggested that "*Commissioners and providers of services to children*

in primary education should develop and agree arrangements to ensure all primary schools adopt a comprehensive whole school approach to children's social and emotional wellbeing. They should provide specific help for those children most at risk (or already showing signs) of social, emotional and behavioural problems" [2]. The challenge facing policy makers and providers is how such a suggestion might be implemented. A useful starting point might arise from linking routine data across health and education services. ASD presents an exemplar condition that could elucidate the challenges and potential utility of data linkage across domains.

Autism Spectrum Disorders are neurodevelopmental disorders that lead to impaired reciprocal social interaction and repetitive or restricted patterns of behaviour [3] occurring in at least 1% of children in the UK [4]. The behavioural problems associated with the condition are a major cause of children being excluded from school [2]. A recent review showed that government policies and community resources impact upon early identification of ASD [5] with evidence of geographic variation. Socio-economic status and level of parental concern affect age of diagnosis [5]. Parents experience high levels of stress with the ASD diagnostic process, with over half dissatisfied with current UK services [6]. On average, families have to wait 3-4 years to receive a diagnosis [6, 7]. Many children are identified early with a range of difficulties but not given a diagnosis of autism until much later [7]. Box 1 provides typical testimony from a parent consulted in our patient and public involvement work (slightly abridged).

Box 1: *We requested a referral to paediatrician as we suspected autism when my son was four years. The SENCO in his nursery had been involved when he was 3.5 years old as staff said he wasn't listening and his attention span was poor. We approached our health visitor after this to express our concerns. They referred him for speech and language therapy. The*

speech and language therapist referred him to the Paediatrician. We waited nearly 2 years to be seen. After an initial appointment we were told they wanted to wait and see. This meant that our son did not get appropriate treatment until he was eight years of age. Should there not be a more systematic way of assessing children in need early?

Early intervention in ASD is associated with long-term symptom reduction [8]. This includes identifying appropriate educational placement early and parenting support interventions [8]. There has been a call for more sophisticated approaches to screening (such as stepped approaches or at risk group identification) since whole population approaches have not proved cost-effective [9].

A universal educational assessment is conducted on *all* children in their first year of schooling in the UK (the Early Years Foundation Stage Profile; EYFSP).We predicted that scores on this developmental assessment might identify children at risk of neurodevelopmental problems. This could lead to earlier intervention to prevent the poor outcomes associated with undiagnosed conditions. Our prediction was motivated by the fact that information collected through the EYFSP routinely covers all the domains of autism symptomatology, providing information about child's language development, social skills and emotional development.

Methods

Design

This was a retrospective data linkage study. To test our hypothesis, we focused on ASD as an exemplar and retrospectively examined the EYFSP scores collected on the 13,500 children within the Born in Bradford longitudinal birth cohort study [10]. This cohort has been followed

up since birth. The BiB cohort comprises 12,453 women recruited at 28 weeks of pregnancy, who gave birth at the Bradford Royal Infirmary to 13,857 children between the period 2007 and 2011 [10]. Half of all BiB families live within wards classed amongst the 20% most deprived within England and Wales, and 45% are of Pakistani origin [10].

Patient and public involvement

A key strength of BiB has been its effective community engagement with considerable time and effort invested to ensure genuine co-production of research. There is an active parent governor group and they advised on the protocol and materials used for this study. BiB has an active approach to partnership with families through regular newsletters, Facebook, and Twitter with regular family network days and an annual family science festival.

Full informed consent was obtained for all participants. Ethical approval was granted by Bradford NHS Research Ethics Committee (Ref 07/H1302/112). Cohort members gave their consent to access GP records via SystmOne, which currently has complete coverage of all GP practices in Bradford. Figure 1 provides an overview of the recruitment process and the final sample included in the current study. Table 1 provides a comparison of the characteristics of the current sample with the whole Born in Bradford cohort.

Confidential: For Review Only

Table 1. Sample characteristics

	Sample in the current study (n = 8,935)	Sample in the whole cohort (n = 13,857)
**Gender**		
Female	49.1%	49.0%
Male	50.9%	51.0%
**Ethnicity**		
White British	36.1%	37.0%
Pakistani Heritage	51.8%	48.6%
Other	12.1%	14.4%
**Free school meal status**		
In receipt	24.7%	25.1%
Not in Receipt	75.3%	74.9%

Outcome measures

Early years foundation stage profile score

Table 2 displays the 17 items of The Early Years Foundation Stage Profile, each scored 2 (meeting the level of development *expected*), 3 (*exceeding* this level) or 1 (not yet reaching this level - *emerging*). The items are designed to measure a range of educational, socio-emotional, communicative and developmental factors. We used the total EYFSP score (the 'total score') and a sub-score developed by a small group of ASD assessment experts prior to study and blind

Confidential: For Review Only

to the results (a 5 item ‘sub-score’) (see table 3). The experts were academic and clinical child psychiatrists and psychologists with many years of ASD experience between them. The five items were chosen from the four main symptom areas defined in the World Health Organisation (1992) [3] research diagnostic criteria for ASD namely social reciprocity, language and communication, imagination delays and repetitive and stereotyped patterns of behaviour. Because the social reciprocity domain is given more weight in this classification system it was decided to include two items from the EYFSP to reflect this weighting.

Sensitivity analysis was carried out using the scores derived from all items.

Read Code Diagnosis of ASD

The outcome measure for this study was the presence of a ‘Read code’ for an ASD recorded in a child’s primary care records. A validated Read code list (see supplementary material 3) has been shown in previous published work in ASD to be reliable and used with confidence to study ASD [11].

Confidential: For Review Only

Table 2 : Early Years Skills Foundation Profile: All items

Physical development: Health and self-care	EYFSP05
Communication and language: Listening and attention	EYFSP01
Personal, social and emotional: Managing feelings and behaviour	EYFSP07
Communication and language: Understanding	EYFSP02
Expressive arts and design: Being imaginative	EYFSP17
Understanding the world: People and communities	EYFSP13
Personal, social and emotional: Self-confidence and self-awareness	EYFSP06
Communication and language: Speaking	EYFSP03
Personal, social and emotional: Making relationships	EYFSP08
Expressive arts and design: Exploring and using media and materials	EYFSP16
Mathematics: Shapes, space and measures	EYFSP12
Physical development: Moving and handling	EYFSP04
Understanding the world: The world	EYFSP14
Mathematics: Numbers	EYFSP11
Literacy: Reading	EYFSP09
Literacy: Writing	EYFSP10
Understanding the world: Technology	EYFSP15

Confidential: For Review Only

**Table 3: Early Years Skills Foundation Profile (EYFSP): Weighted sub-score**
Using a weighted sub-score, where four aspects of childhood autism (social, language and communications, imagination and repetitive behaviour) were mapped onto EYFSP elements
**The social aspect mapped onto:**
• Personal, social and emotional: Managing feelings and behaviour/
• Personal, social and emotional: Making relationships
**Language and communications aspect mapped onto:**
• Communication and language: Listening and attention
**Imagination aspect mapped onto:**
• Expressive arts and design: Being imaginative
**Repetitive and stereotyped behaviours mapped onto:**
• Physical development: Health and self-care

Confidential: For Review Only

Procedure

Linkage was carried out using NHS number, surname and date of birth (99% of children matched) and education records. At the point of analysis, 8, 935 children were included.

Analysis

Logistic regression was employed to model the relationship between EYFSP score and the outcome of ASD diagnosis using Stata 13 [12]. A number of covariates were included in the analysis; gender (classified as male or female), ethnicity (White British, Pakistani Heritage and Other), free school meal status (whether in receipt of free school meals or not) and the age of the child (in years) at the date of GP data extract. Marginal effects were estimated [13] to produce predicted rates of ASD diagnosis for children based on whether they had a low EYFSP score.

The total EYFSP score ranged from 17 to 51, with a mean of 31.8 and a standard deviation of 8.0. The five item sub-score ranged from 7 to 21, with a mean of 13.6 and a standard deviation of 3.4 (see figure 2). However, the EYFSP scores are not normally distributed; mostly children score average on each item, but there are some who score higher and a fairly distinct group of children who have low scores resulting in a bimodal distribution of EYFSP scores, as indicated in figure 3. For this reason the EYFSP scores are dichotomised for the purposes of analysis. Those scoring below 25 in the total EYFSP score and below 10 in the five item sub-score were categorised as low scores; these cut points equate to scores less than one standard deviation below the mean (see figure 3).

Confidential: For Review Only

Confidential: For Review Only

Results

We found that children with a low EYFSP sub-score were approximately 50 times more likely to have a diagnosis of autism compared to children without a low score. Males were twice as likely to have a diagnosis of ASD, whilst White British children and children not in receipt of free school meals had higher rates of diagnosis recorded in the GP records, apart from age of the child, all differences were statistically significant and all covariates improved the model (see supplementary material 1 for full model results). Sensitivity analysis was carried out using the scores derived from all items and produced similar results; though the association was stronger for the EYFSP sub-scores (see Figure 4).

Children with a low total EYFSP score (A) have a rate of autism of around 41 per 1,000 children (4.10%, 95% CI: 3.08 - 5.13), far higher than children who do not have a low total EYFSP score (0.15%, 95% CI: 0.05 - 0.25).

Children with a low five item EYFSP sub-score (B) have a rate of autism of around 64 per 1,000 children (6.38%, 95% CI: 4.76 - 8.00), far higher than children who do not have a low five item EYFSP sub-score (0.12%, 95% CI: 0.04 - 0.20).

Discussion

Our linkage of routine education and health data has revealed an association between school EYFSP score profile and the diagnosis of ASD. We focused this proof-of-concept study on ASD as: (i) the issues with late diagnosis are well documented; (ii) early diagnosis is known to lead to enhanced parental interventions and (iii) improved educational pathways lead to better outcomes. The linkage process and subsequent analyses of the linked datasets has illustrated a potentially

powerful way to create a smoother, more joined-up and timely early patient pathway to autism assessment by linking data across the currently tortuous pathway to referral for ASD assessment. Gold standard assessment for ASD is lengthy and expensive [14] so care needs to be taken to ensure that screening has good specificity and sensitivity to avoid large numbers of unnecessary assessments. Further research is therefore necessary. Population screening for ASD is also expensive [14] and to date, the effectiveness of different screening strategies for ASD in reducing time to diagnosis has not been adequately tested [9]. Whilst researchers have recommended that screening for ASD only be carried out in 'at risk' groups [15], no localities or nations have yet successfully implemented or researched this. Our study suggests that a promising focus for future research lies in the use of routine educational data to identify children at risk. This could be highly cost-effective given the universal availability of this educational data.

Related school based information is often collected as part of the assessment for neurodevelopment disorders (including ASD). Consequently correlation between EYFSP scores and neurodevelopmental disorders may be expected. Routinely collected national school data may therefore be a cost effective way of initially identifying children at risk, however larger studies are required to adequately test this across neurodevelopmental disorders.

Clinical and Research Implications

Encouraged by strong stakeholder support, we are now prospectively researching whether children identified by the EYFSP is a suitable 'at risk' group for specific ASD screening and assessment for ASD in terms of acceptability, ethics and cost-effectiveness, and whether it is a sensitive and specific method of enabling prompt assessment leading to early intervention.

If successful it would substantially increase efficiencies in the delivery of current services. These include assessment resources and appropriate school placement/support earlier. It may also prevent multiple assessments from different professionals over time. We have recently shown an inequality of diagnosis as a function of socioeconomic position [16] and the use of routine linked health and education data could be a powerful tool to help tackle such health inequalities.

Routine electronic data that are relevant to health and care pathways exist in fragments, spread across multiple organisations, each fragment with its own data controller. The challenges of cross-organisation coordination and information governance make it difficult to obtain linked data, and so most care providers and researchers have been unable to use it as a means of understanding whole-system healthcare. In Bradford, we have addressed this as part of the Connected Health Cities project, by reaching data sharing agreements with each of the health and social care organisations in the Bradford city region. Identifiable information is removed at source, and so personal information is not available to the project, which enables whole population data to be analysed. The pseudonym is derived from the NHS number, which crosses organisations, so that data can be linked after pseudonymisation. School UPN numbers are input into the electronic health record by the school nurse service. There is good scope for this approach to be used across multiple Trusts.

Conclusions

Our 'proof-of-concept' study suggests that linking education and health data could improve the detection and support of children with neurodevelopmental problems such as ASD earlier – a priority area identified by the Chief Medical Officer [2]. The use of linked data to benefit outcomes is an important future goal [17]. In this context our demonstration has implications beyond autism. It is becoming clear that the routine linkage of education and health data has the

Confidential: For Review Only

potential to drive efficiencies in children's services, facilitate early intervention and ultimately, improve quality of life for large numbers of children and their families.

Acknowledgments

Acknowledgements: Connected Health Cities is a Northern Health Science Alliance led programme funded by the Department of Health and delivered by a consortium of academic and NHS organisations across the north of England. The work uses data provided by patients and collected by the NHS as part of their care and support. The views expressed are those of the author(s) and not necessarily those of the NHSA, NHS or the Department of Health. We would like to acknowledge the support of Jane Blackwell and Kuldeep Sohal.

Funding: This research received no specific grant from any funding agency in the public, commercial or not-for-profit sectors.

Competing interests: The authors have declared that no competing interests exist.

Authors contributions: Contributed to the writing of the manuscript: BW, MM-W, BK, SW, DS, FM, JW. Statistical analysis BK, Agree with the manuscript's results and conclusions: BW, MM-W, BK, SW, DS, FM, JW. All authors have read, and confirm that they meet, ICMJE criteria for authorship.

Abbreviations: ASD - Autism Spectrum Disorders; CMO – Chief Medical Officer; EYFSP – Early Years Foundation Stage Profile Score; NHS – National Health Service;

Provenance: Not commissioned; externally peer-reviewed

Data sharing statement: The Born in Bradford (BiB) dataset is available from: https://borninbradford.nhs.uk/research/how-to-access-data/

Confidential: For Review Only

References

1. NICE Local Government Briefings. Social and emotional wellbeing for children and young people. 25 September 2013.

2. Chief Medical Officer's annual report 2012: Our Children Deserve Better: Prevention Pays (published Oct 2013)

3. World Health Organization. The ICD-10 classification of mental and behavioural disorders: diagnostic criteria for research, 1992.

4. Baird, G., Simonoff, E., Pickles, A., Chandler, S., Loucas, T., Meldrum, D. and Charman, T. Prevalence of disorders of the autism spectrum in a population cohort of children in South Thames: the Special Needs and Autism Project (SNAP). *Lancet* 2006; 368(9531): 210-215.

5. Daniels AM, Mandell D. Explaining differences in age at autism spectrum disorder diagnosis: A critical review. *Autism* 2013; 18(5): 583-597.

6. Crane L, Chester JW, Goddard L, Henry LA, Hill E. Experiences of autism diagnosis: A survey of over 1000 parents in the United Kingdom. Autism 2016; 20(2): 153-162.

7. Care Quality Commission. Health Care for Disabled Children and Young People. 2012.

8. Pickles A, Le Couteur A, Leadbitter K, Salomone E, Cole-Fletcher R, Tobin H, Gammer I, Lowry J, Vamvakas G, Byford S, Aldred C. Parent-mediated social communication therapy for young children with autism (PACT): long-term follow-up of a randomised controlled trial. *The Lancet*, 2016; 388(10059): 2501-2509.

9. Daniels AM, Halladay AK, Shih A, Elder LM, Dawson G. Approaches to enhancing the early detection of autism spectrum disorders: a systematic review of the literature. *Journal of the American Academy of Child & Adolescent Psychiatry,* 2014; 53(2): 141-152.

10. Wright J, Small N, Raynor P, Tuffnell D, Bhopal R, Cameron N, Fairley L, Lawlor DA, Parslow R, Petherick ES.
and Pickett KE.. Cohort Profile: the Born in Bradford multi-ethnic family cohort study. *International Journal of Epidemiology* 2013; 42(4): 978–91.

11. Hagberg, K,W., & Jick, S.S. Validation of autism spectrum disorder diagnoses recorded in the Clinical Practice Research Datalink, 1990–2014. *Clinical Epidemiology*, 2017; *9*, p.475

12. StataCorp. Stata Statistical Software: Release 13. College Station, Texas: StataCorp LP: 2013.

13. Williams R. Using the margins command to estimate and interpret adjusted predictions and marginal effects. *Stata Journal* 2012; 12: 308–331.

Confidential: For Review Only

14. Falkmer T, Anderson K, Falkmer M, Horlin C. Diagnostic procedures in autism spectrum disorders: a systematic literature review. *European child & adolescent psychiatry* 2013; 22(6): 329-340.

15. Towle PO, Patrick PA. Autism Spectrum Disorder Screening Instruments for Very Young Children: A Systematic Review. *Autism Research and Treatment*, 2016.

16. Kelly B, Williams S, Collins S, Mushtaq F, Mon-Williams M, Wright B, Mason D, Wright J. The association between socioeconomic status and autism diagnosis in the United Kingdom for children aged 5–8 years of age: Findings from the Born in Bradford cohort. *Autism* 2017; Nov 7:1362361317733182.

17. Schneeweiss S. Learning from big health care data. *New England Journal of Medicine* 2014; 370(23): 2161-2163.

Figure legends

Figure 1: Recruitment flow chart

Figure 2: Early Years Foundation Stage Profile (EYFSP): Weighted sub-score

Figure 3: Distribution of EYFSP Scores - standardised with standard deviation shown

Figure 4: Predicted rates of ASD for children by (A) total EYFSP score and (B) five item EYFSP sub-score (designed to map more closely to items that measure factors associated with autism)

Confidential: For Review Only

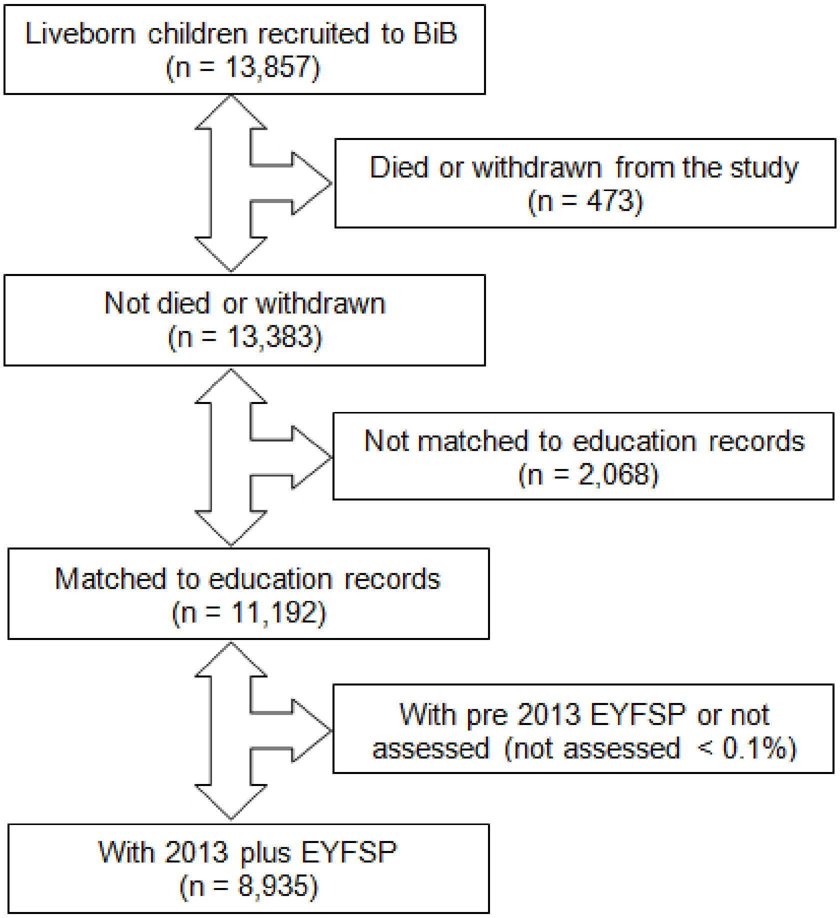
Liveborn children recruited to BiB
(n = 13,857)
Died or withdrawn from the study
(n = 473)
Not died or withdrawn
(n = 13,383)
Not matched to education records
(n = 2,068)
Matched to education records
(n = 11,192)
With pre 2013 EYFSP or not assessed (not assessed < 0.1%)
With 2013 plus EYFSP
(n = 8,935)

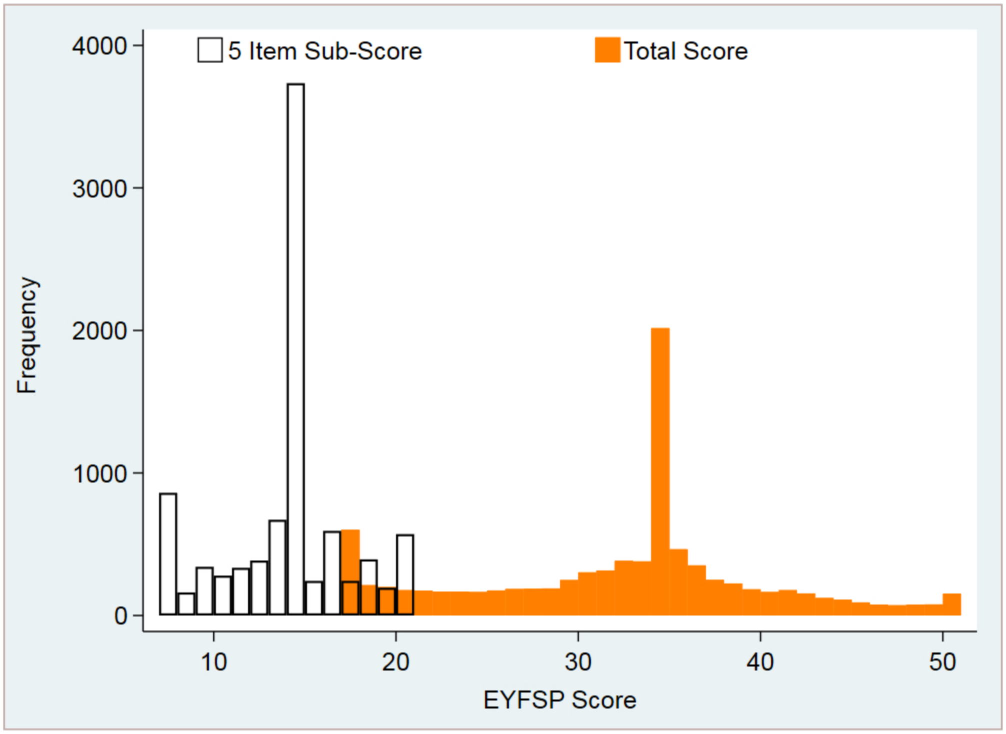

352x256mm (72 x 72 DPI)

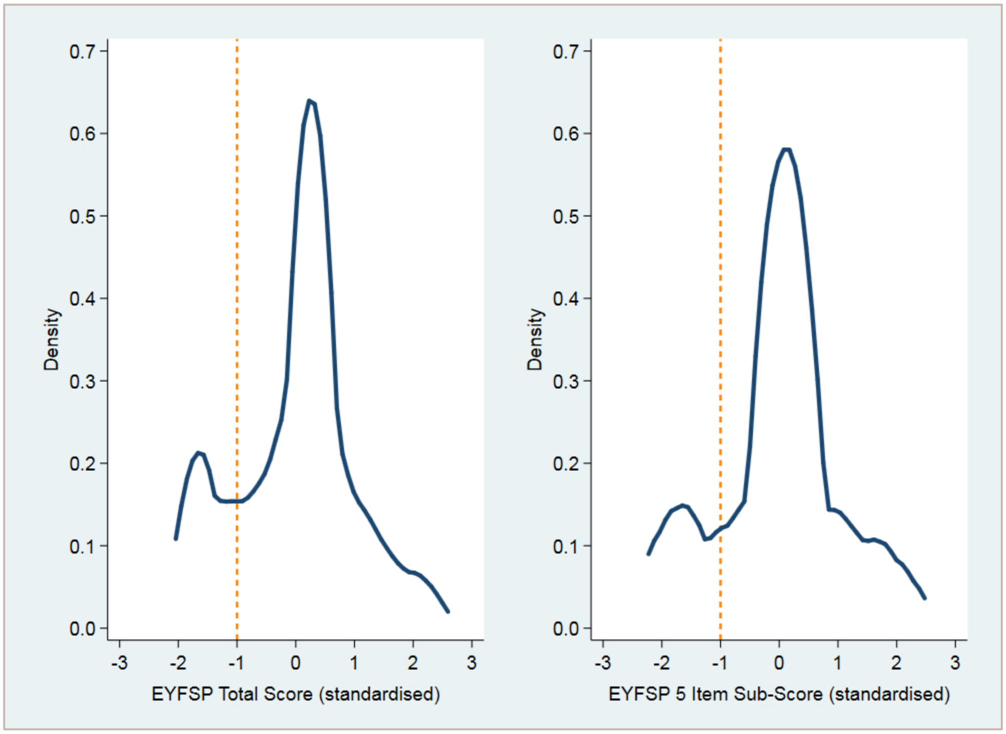

352x256mm (72 x 72 DPI)

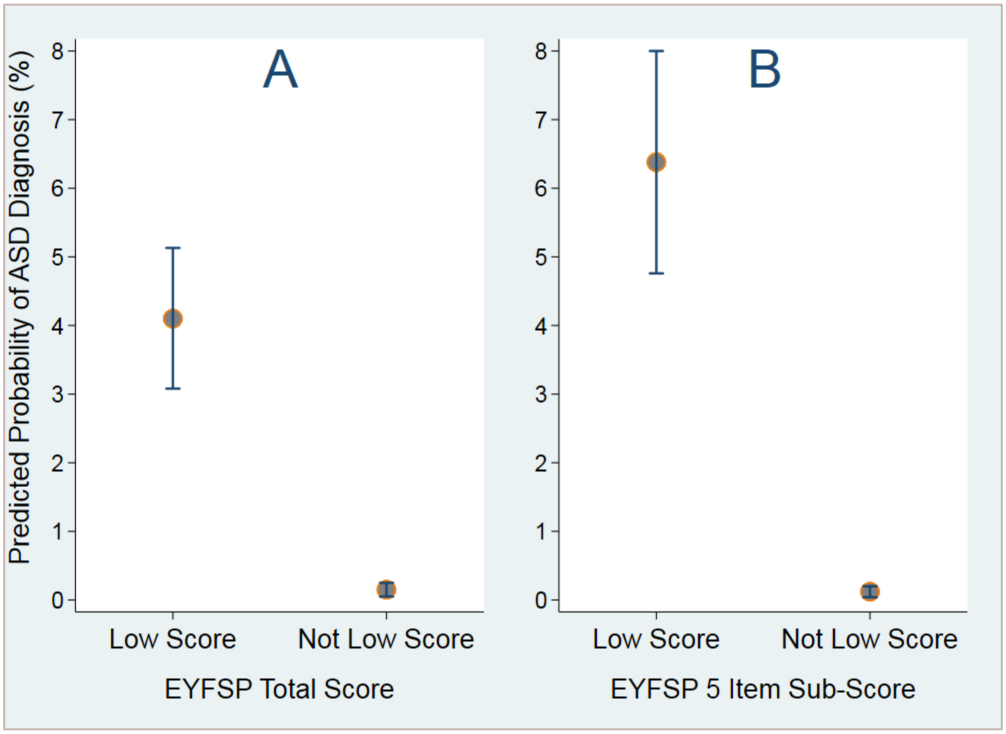

352x256mm (72 x 72 DPI)

Supplementary material 1: Results of logistic regression models

Logistic Regression Models	EYFSP Total Score			EYFSP 5 Item Sub-Score		
	Odds Ratio	95% Confidence Intervals Low	High	Odds Ratio	95% Confidence Intervals Low	High
**Univariate: EYFP score**						
Not low EYSPF score (reference)						
Low EYSFP score	23.7*	12.9	43.8	39.9*	21.6	73.8
**Multivariate: EYFP score and covariates**						
Not low EYSPF score (reference)						
Low EYSFP score	29.4*	14.4	59.8	58.1*	27.4	123.0
Gender: Female (reference)						
Gender: Male	2.87*	1.56	5.29	2.38*	1.28	4.41
Child ethnicity: White British (reference)						
Pakistani Heritage	0.45*	0.27	0.76	0.42*	0.24	0.71
Other	0.69	0.33	1.41	0.63	0.30	1.32
Receiving free school meals (reference)						
Not receiving free school meals	2.29*	1.25	4.19	2.39*	1.30	4.41
Age (years) at GP data extract: Five (reference)						
Six	1.60	0.54	4.76	1.16	0.38	3.54
Seven	0.94	0.31	2.80	0.67	0.22	2.05
Eight plus	0.93	0.31	2.82	0.71	0.23	2.19

* = significant at the 0.05 level

Confidential: For Review Only

Supplementary material 2: Kernel density of the total score and the weighted sub-score

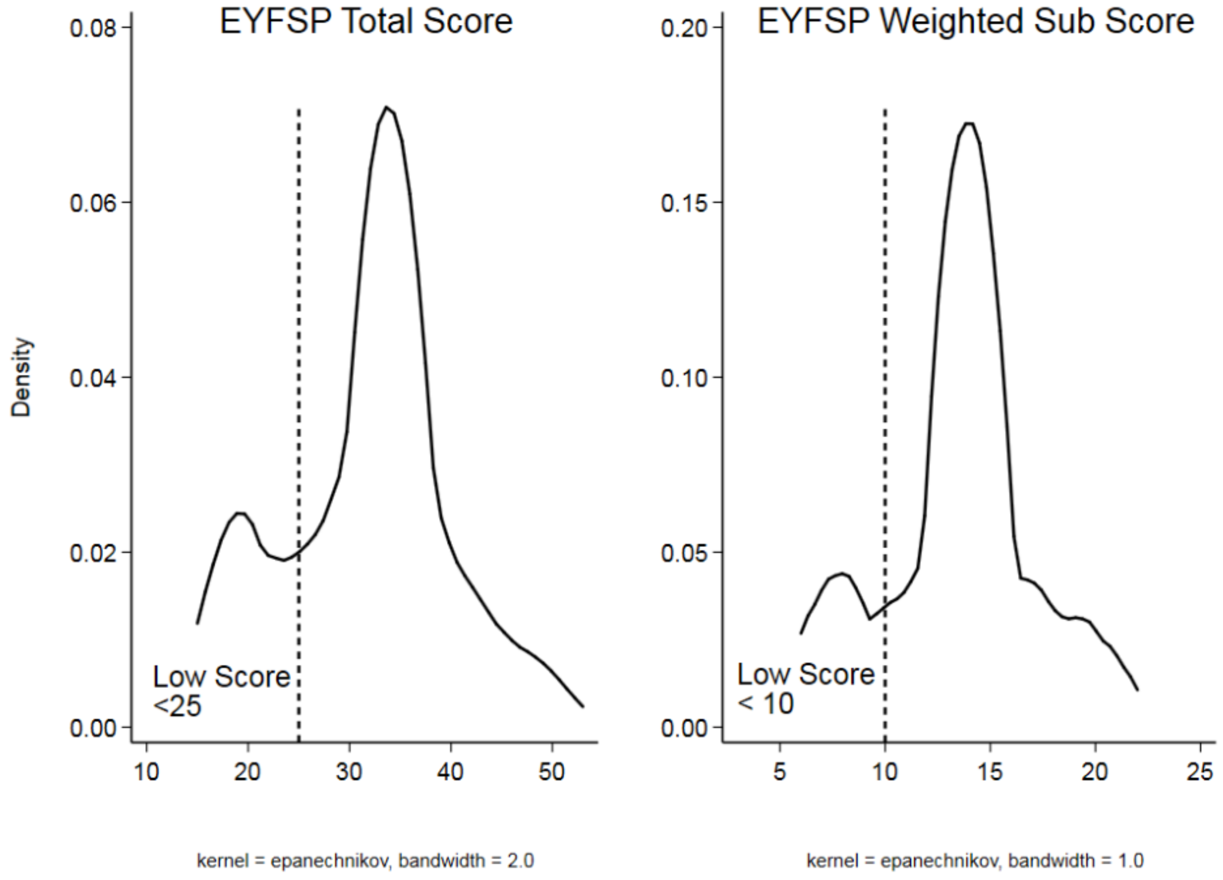

Supplementary material 3: Read codes for autism

We used the Read codes below to identify children with a diagnosis of autism. This was supplemented by a free text search (which did not identify further cases).

**Read code**	**Read code description**
**ASDs**	
E140.00	Infantile autism
E140000	Active infantile autism
E140100	Residual infantile autism
E140.12	Autism[**]
E140.13	Childhood autism
E140000	Active infantile autism
E140z00	Infantile autism NOS
Eu84000	[X]Childhood autism
Eu84011	[X]Autistic disorder[**]
Eu84012	[X]Infantile autism
Eu84100	[X]Atypical autism
Eu84z11	[X]Autistic spectrum disorder
Eu84500	[X]Asperger's syndrome[**]

Confidential: For Review Only

BMJ Paediatrics Open

Investigating the association between early years foundation stage profile scores and subsequent diagnosis of an autism spectrum disorder: A retrospective study of linked healthcare and education data

Journal:	*BMJ Paediatrics Open*
Manuscript ID	bmjpo-2019-000483.R2
Article Type:	Original article
Date Submitted by the Author:	06-Sep-2019

Complete List of Authors:	Wright, Barry; University of York, Health Sciences<br>Mon-Williams, Mark; University of Leeds, Institute of Psychological Sciences<br>Kelly, Brian; Bradford Institute for Health Research<br>Williams, Stefan; Bradford Teaching Hospitals NHS Foundation Trust<br>Sims, David; Bradford District Care NHS Foundation Trust<br>Mushtaq, Faisal; University of Leeds, Institute of Psychological Sciences<br>Sohal , Kuldeep; Bradford Institute for Health Research<br>Blackwell , Jane; Leeds and York Partnership NHS Foundation Trust, Child Oriented Mental health Intervention Centre<br>Wright, Prof John; Bradford Teaching Hospitals NHS Foundatin Trust, Bradford Institute for Health Research

Keywords:	Autism, Screening, Data Collection, Health Service

SCHOLARONE™
Manuscripts

Investigating the association between early years foundation stage profile scores and subsequent diagnosis of an autism spectrum disorder: A retrospective study of linked healthcare and education data

Barry Wright[1, 2], Mark Mon-Williams[3,4], Brian Kelly[4], Stefan Williams[5], David Sims[6], Faisal Mushtaq[3], Kuldeep Sohal[4], Jane Blackwell[2], John Wright[4]

Affiliations:

1. Hull York Medical School, University of York, UK
2. Leeds and York Partnership NHS Foundation Trust
3. School of Psychology, University of Leeds, UK
4. Bradford Institute for Health Research, Bradford, UK
5. Bradford Hospitals NHS Teaching Hospitals NHS Foundation Trust
6. Bradford District Care NHS Foundation Trust

Corresponding author

Professor Barry Wright

Hull York Medical School

University of York

York

YO10 5DD

UK

Email: Barry.wright1@nhs.net

Telephone number: 01904 724540

Confidential: For Review Only

Abstract

Objective: We set out to test whether the Early Years Foundation Stage Profile score derived from 17 items assessed by teachers at the end of reception school year had any association with Autism Spectrum Disorders diagnosis in subsequent years. This study tested the feasibility of successfully linking education and health data.

Design: A retrospective data linkage study.

Setting and participants: The Born in Bradford longitudinal cohort of 13,857 children.

Outcome measures: We linked the Early Years Foundation Stage Profile (EYFSP) score at the end of reception year with subsequent diagnosis of an autism spectrum disorder, using all ASD general practitioner Read codes. We used the total EYFSP score and a sub-score consisting of 5 key items in the EYFSP, prospectively identified using a panel of early years autism experts.

Results: This study demonstrated the feasibility of linking education and health data using Autism Spectrum Disorders as an exemplar. A total of 8,935 children had linked GP and education data with 20.7% scoring below 25 on the total EYFSP and 15.2% scoring below 10 on a EYFSP sub-score proposed by an expert panel prospectively. The rate of diagnosis of Autism Spectrum Disorders at follow up just under 1% (84 children), children scoring below 25 on the total EYFSP had a 4.1% chance of ASD compared to 0.15% of the remaining children. Using the prospectively designed sub-score this difference was greater (6.4% and 0.12% respectively).

Conclusions: We demonstrate the feasibility of linking education and health data. Performance on teacher ratings taken universally in school reception class can flag children at risk of autism spectrum disorders. Further research is warranted to explore the utility of EYFSP as an initial screening tool for ASD in early school years.

What is already known about this topic?

- Routine education and health data are rarely linked.
- To date there is limited evidence that screening for autism spectrum disorders in the first 6

Confidential: For Review Only

What this study adds?

- It is feasible to link routine education and health data for a cohort of children in England.
- Performance on educational measures taken universally in school reception class can flag children at risk of autism spectrum disorders.
- Linking these data has the potential to decrease costs associated with undiagnosed childhood

This is an Open Access article distributed in accordance with the Creative Commons Attribution Non Commercial Licence (CC BY NC – 4.0) which permits others to distribute and build on this work non-commercially, provided the original work is properly cited and the use is non-commercial.

Introduction

Large quantities of electronic data are generated routinely in health and education sectors. Linking these data could present an opportunity to improve information sharing and facilitate service user transitions within and across systems.

10% of children and young people aged 5–16 years have a clinically diagnosed mental disorder [1]. The short-term costs are estimated to be £1.58 billion per annum, with annual long-term

costs calculated at £2.35 billion [2]. Neurodevelopmental impairments and conditions with high levels of need contributing to this cost have an estimated prevalence of around 3–4% of children in England [2]. The CMO suggested that "*Commissioners and providers of services to children in primary education should develop and agree arrangements to ensure all primary schools adopt a comprehensive whole school approach to children's social and emotional wellbeing. They should provide specific help for those children most at risk (or already showing signs) of social, emotional and behavioural problems*" [2]. The challenge facing policy makers and providers is how such a suggestion might be implemented. A useful starting point might arise from linking routine data across health and education services. ASD presents an exemplar condition that could elucidate the challenges and potential utility of data linkage across domains.

Autism Spectrum Disorders (ASD) are neurodevelopmental disorders that lead to impaired reciprocal social interaction and repetitive or restricted patterns of behaviour [3] occurring in at least 1% of children in the UK [4]. The behavioural problems associated with the condition are a major cause of children being excluded from school [2]. A recent review showed that government policies and community resources impact upon early identification of ASD [5] with evidence of geographic variation. Socio-economic status and level of parental concern affect age of diagnosis [5]. Parents experience high levels of stress with the ASD diagnostic process, with over half dissatisfied with current UK services [6]. On average, families have to wait 3-4 years to receive a diagnosis [6, 7]. Many children are identified early with a range of difficulties but not given a diagnosis of autism until much later [7]. Box 1 provides typical testimony from a parent consulted in our patient and public involvement work (slightly abridged).

Box 1: *We requested a referral to paediatrician as we suspected autism when my son was four years. The SENCO in his nursery had been involved when he was 3.5 years old as staff said he wasn't listening and his attention span was poor. We approached our health visitor after this to express our concerns. They referred him for speech and language therapy. The speech and language therapist referred him to the Paediatrician. We waited nearly 2 years to be seen. After an initial appointment we were told they wanted to wait and see. This meant that our son did not get appropriate treatment until he was eight years of age. Should there not be a more systematic way of assessing children in need early?*

Confident

Early intervention in ASD is associated with long-term symptom reduction [8]. This includes identifying appropriate educational placement early and parenting support interventions [8]. There has been a call for more sophisticated approaches to screening (such as stepped approaches or at-risk group identification) since whole population approaches have not proved cost-effective [9].

A universal educational assessment is conducted on *all* children in their first year of schooling in the UK (the Early Years Foundation Stage Profile; EYFSP). We predicted that scores on this developmental assessment might identify children at risk of neurodevelopmental problems. This could lead to earlier intervention to prevent the poor outcomes associated with undiagnosed conditions. Our prediction was motivated by the fact that information collected through the EYFSP routinely covers key domains of autism symptomatology, providing information about child's language development, social skills and emotional development.

Methods

Design

This was a retrospective data linkage study. To test our hypothesis, we focused on ASD as an exemplar and retrospectively examined the EYFSP scores collected on the 13,857 children within the Born in Bradford (BiB) longitudinal birth cohort study [10]. This cohort has been followed up since birth. The BiB cohort comprises 12,453 women recruited at 28 weeks of pregnancy, who gave birth at the Bradford Royal Infirmary to 13,857 children between the period 2007 and 2011 [10]. Half of all BiB families live within wards classed amongst the 20% most deprived within England and Wales, and 45% are of Pakistani origin [10].

Patient and public involvement

A key strength of BiB has been its effective community engagement with considerable time and effort invested to ensure genuine co-production of research. There is an active parent governor group and they advised on the protocol and materials used for this study. BiB has an active approach to partnership with families through regular newsletters, Facebook, and Twitter with regular family network days and an annual family science festival.

Full informed consent was obtained for all participants. Ethical approval was granted by Bradford NHS Research Ethics Committee (Ref 07/H1302/112). Cohort members gave their consent to access and link routine GP records via SystmOne, which currently has complete coverage of all GP practices in Bradford with secondary care records and education records. Figure 1 provides an overview of the recruitment process and the final sample included in the current study. Table 1 provides a comparison of the characteristics of the current sample with the whole Born in Bradford cohort.

Confidential: For Review Only

Confidential: For Review Only

Table 1. Sample characteristics

	**Sample in the current study** (n = 8,935)	**Sample in the whole cohort** (n = 13,857)
**Gender**		
Female	49.1%	49.0%
Male	50.9%	51.0%
**Ethnicity**		
White British	36.1%	37.0%
Pakistani Heritage	51.8%	48.6%
Other	12.1%	14.4%
**Free school meal status**		
In receipt	24.7%	25.1%
Not in Receipt	75.3%	74.9%

Outcome measures

Early years foundation stage profile score

Table 2 displays the 17 items of The Early Years Foundation Stage Profile, each scored 2 (meeting the level of development *expected*), 3 (*exceeding* this level) or 1 (not yet reaching this

level - *emerging*). The items are designed to measure a range of educational, socio-emotional, communicative and developmental factors. We used the total EYFSP score (the ‘total score’) and a sub-score developed by a small group of ASD assessment experts prior to study (and so blind to the results) (a 5 item ‘sub-score’) (see Table 3). The experts were academic and clinical child psychiatrists and psychologists with many years of ASD experience between them. The five items were chosen from the four main symptom areas defined in the World Health Organisation (1992) [3] research diagnostic criteria for ASD namely social reciprocity, language and communication, imagination delays and repetitive and stereotyped patterns of behaviour. Because the social reciprocity domain is given more weight in this classification system it was decided to include two items from the EYFSP to reflect this weighting. Children who underwent EYFSP assessment before 2013 were excluded from this study because the items measured in the EYFSP assessment were changed in 2013. The pre-2013 version is not compatible with the current EYFSP assessment.

Read Code Diagnosis of ASD

The outcome measure for this study was the presence of a ‘Read code’ for an ASD recorded in a child’s primary care records. A validated Read code list (see Supplementary Material 2) has been shown in previous published work in ASD to be reliable and used with confidence to study ASD [11].

Confidential: For Review Only

Table 2: Early Years Skills Foundation Profile: All items

Physical development: Health and self-care	EYFSP05
Communication and language: Listening and attention	EYFSP01
Personal, social and emotional: Managing feelings and behaviour	EYFSP07
Communication and language: Understanding	EYFSP02
Expressive arts and design: Being imaginative	EYFSP17
Understanding the world: People and communities	EYFSP13
Personal, social and emotional: Self-confidence and self-awareness	EYFSP06
Communication and language: Speaking	EYFSP03
Personal, social and emotional: Making relationships	EYFSP08
Expressive arts and design: Exploring and using media and materials	EYFSP16
Mathematics: Shapes, space and measures	EYFSP12
Physical development: Moving and handling	EYFSP04
Understanding the world: The world	EYFSP14
Mathematics: Numbers	EYFSP11
Literacy: Reading	EYFSP09
Literacy: Writing	EYFSP10
Understanding the world: Technology	EYFSP15

Confidential: For Review Only

**Table 3: Early Years Skills Foundation Profile (EYFSP): Weighted sub-score**
Using a weighted sub-score, where four aspects of childhood autism (social, language and communications, imagination and repetitive behaviour) were mapped onto EYFSP elements
**The social aspect mapped onto:**
• Personal, social and emotional: Managing feelings and behaviour/
• Personal, social and emotional: Making relationships
**Language and communications aspect mapped onto:**
• Communication and language: Listening and attention
**Imagination aspect mapped onto:**
• Expressive arts and design: Being imaginative
**Repetitive and stereotyped behaviours mapped onto:**
• Physical development: Health and self-care

Confidential: For Review Only

Confidential: For Review Only

Procedure

In collaboration with our primary care electronic health provider across Bradford, linkage was carried out using a complete deterministic match on NHS number, surname, gender and date of birth (99% of BiB children matched to their health record) by the BiB data team. Routine electronic datasets include primary care data from the GP practice, hospital data, community care and education data. Hospital and maternity data was provided by Bradford Teaching Hospitals NHS Foundation Trust. The community care data was provided by Bradford District Care Trust. In collaboration with our local provider of electronic health records across primary care, the GP records were extracted by The Phoenix Partnership, SystmOne. The education dataset was provided by Bradford Metropolitan District Council. Our local authority, Bradford Metropolitan District Council linked BiB children to their Unique Pupil Identification Number (UPN) resulting in a match rate of 84% with their education records. The Unique Pupil Identification Number is a 13-character code that identifies each pupil in the local-authority school system. As the local authority match on an iterative deterministic approach based on combinations of surname, date of birth, gender and postcode, if more than one match is made, then we do not receive any UPNs. At the point of analysis, 8,935 children were included. 2068 children could not be linked mainly because the children were born in Bradford but moved outside the area

(approximately 1,200 children) or had moved in after EYFSP (approximately 200) children. The other reason for exclusion was an inability to match to pre-determined quality standards.

Analysis

Logistic regression was employed to model the relationship between EYFSP score and the outcome of ASD diagnosis using Stata 13 [12]. A number of covariates were included in the analysis; gender (classified as male or female), ethnicity (White British, Pakistani Heritage and Other), free school meal status (whether in receipt of free school meals or not) and the age of the child (in years) at the date of GP data extract. Marginal effects were estimated [13] to produce predicted rates of ASD diagnosis for children based on whether they had a low EYFSP score.

The total EYFSP score ranged from 17 to 51, with a mean of 31.8 and a standard deviation of 8.0. The five item sub-score ranged from 7 to 21, with a mean of 13.6 and a standard deviation of 3.4 (see Figure 2). However, the EYFSP scores are not normally distributed; mostly children score average on each item, but there are some who score higher and a fairly distinct group of children who have low scores resulting in a bimodal distribution of EYFSP scores, as indicated in figure 3. For this reason, the EYFSP scores are dichotomised for the purposes of analysis. Those scoring below 25 in the total EYFSP score and below 10 in the five item sub-score were categorised as low scores; these cut points equate to scores less than one standard deviation below the mean (see Figure 3).

Results

Confidential: For Review Only

Just under 1% (84 children) of the 8,935 children with matched GP and education records had a diagnosis of autism. Of the 8,935 children had linked GP and education data. 1,852 (20.7%) children scored below the cut off on the total EYFSP score, 72 of these children had an autism diagnosis. 1,355 (15.2%) children scored below the cut off on the EYFSP sub score, 72 of these children had an autism diagnosis (see Table 4).

Table 4: Autism diagnosis and EYFSP scores

	Total EYFSP Score		
	Low Score	Not Low Score	Total
With Autism Diagnosis	72	12	84
No Autism Diagnosis	1,780	7,071	8,851
Total	1,852	7,083	8,935

	EYFSP Sub-Score		
	Low Score	Not Low Score	Total
With Autism Diagnosis	72	12	84
No Autism Diagnosis	1,283	7,568	8,851
Total	1,355	7,580	8,935

We found that children with a low EYFSP sub-score were approximately 50 times more likely to have a diagnosis of autism compared to children without a low score. Males were twice as likely to have a diagnosis of ASD, whilst White British children and children not in receipt of free school meals had higher rates of diagnosis recorded in the GP records, apart from age of the

https://mc.manuscriptcentral.com/bmjpo

Confidential: For Review Only

child, all differences were statistically significant and all covariates improved the model (see Supplementary Material 1 for full model results). Analysis was carried out using the scores derived from all items and produced similar results; though the association was stronger for the EYFSP sub-scores (see Figure 4).

Children with a low total EYFSP score (A) have a rate of autism of around 41 per 1,000 children (4.10%, 95% CI: 3.08 - 5.13), far higher than children who do not have a low total EYFSP score (0.15%, 95% CI: 0.05 - 0.25).

Children with a low five item EYFSP sub-score (B) have a rate of autism of around 64 per 1,000 children (6.38%, 95% CI: 4.76 - 8.00), far higher than children who do not have a low five item EYFSP sub-score (0.12%, 95% CI: 0.04 - 0.20).

Confidential: For

Discussion

Our linkage of routine education and health data has revealed an association between school EYFSP score profile and the diagnosis of ASD. We focused this proof-of-concept study on ASD as: (i) the issues with late diagnosis are well documented; (ii) early diagnosis is known to lead to enhanced parental interventions; and (iii) improved educational pathways lead to better outcomes. The linkage process and subsequent analyses of the linked datasets has illustrated a potentially powerful way to create a smoother, more joined-up and timely early patient pathway to autism assessment by linking data across the currently tortuous pathway to referral for ASD assessment.

Gold standard assessment for ASD is lengthy and expensive [14] so care needs to be taken to ensure that screening has good specificity and sensitivity to avoid large numbers of unnecessary assessments. Further research is therefore necessary. Population screening for ASD is also

expensive [14] and to date, the effectiveness of different screening strategies for ASD in reducing time to diagnosis has not been adequately tested [9]. Whilst researchers have recommended that screening for ASD only be carried out in 'at risk' groups [15], no localities or nations have yet successfully implemented or researched this. Our study suggests that a promising focus for future research lies in the use of routine educational data to identify children at risk. This could be highly cost-effective given the universal availability of this educational data.

Related school-based information is often collected as part of the assessment for neurodevelopment disorders (including ASD). Consequently, correlation between EYFSP scores and neurodevelopmental disorders may be expected. Routinely collected national school data may therefore be a cost-effective way of initially identifying children at risk, however larger studies are required to adequately test this across neurodevelopmental disorders.

Clinical and Research Implications

Encouraged by strong stakeholder support, we are now prospectively researching whether children identified by the EYFSP is a suitable 'at risk' group for specific ASD screening and assessment for ASD in terms of acceptability, ethics and cost-effectiveness, and whether it is a sensitive and specific method of enabling prompt assessment leading to early intervention.

If successful it would substantially increase efficiencies in the delivery of current services. These include assessment resources and appropriate school placement/support earlier. It may also prevent multiple assessments from different professionals over time. We have recently shown an inequality of diagnosis as a function of socioeconomic position [16] and the use of routine linked health and education data could be a powerful tool to help tackle such health inequalities.

Routine electronic data that are relevant to health and care pathways exist in fragments, spread across multiple organisations, each fragment with its own data controller. The challenges of cross-organisation coordination and information governance make it difficult to obtain linked data, and so most care providers and researchers have been unable to use it as a means of understanding whole-system healthcare. In Bradford, we have addressed this as part of The Connected Bradford Project, by reaching data sharing agreements with each of the health and social care organisations in the Bradford city region. Identifiable information is removed at source, and so personal information is not available to the project, which enables whole population data to be analysed. The pseudonym is derived from the NHS number, which crosses organisations, so that data can be linked after pseudonymisation. The Connected Bradford project at Bradford Teaching Hospitals NHS Foundation Trust (BTHFT) applied to the Confidentiality Advisory Group (CAG) for approval under Regulation 5 of the Health Service (Control of Patient Information) Regulations 2002 to process confidential patient information without consent. Approved applications enable the data controller to provide specified information for the purposes of the relevant activity, without being in breach of the common law duty of confidentiality, although other relevant legislative provisions will still be applicable. Support was granted on the 3rd September 2018 (CAG ref: 18/CAG/0091 and REC ref:18/YH/0200). BTHFT have permission under these regulations to provide personal details to the local authority to enable the linkage of education records for approximately 220,000 individuals of the Bradford population. There is good scope for this approach to be used across multiple Trusts.

Conclusions

Confidential: For Review Only

Our ‘proof-of-concept’ study suggests that linking education and health data could improve the detection and support of children with neurodevelopmental problems such as ASD earlier – a priority area identified by the Chief Medical Officer [2]. The use of linked data to benefit outcomes is an important future goal [17]. In this context our demonstration has implications beyond autism. It is becoming clear that the routine linkage of education and health data has the potential to drive efficiencies in children’s services, facilitate early intervention and ultimately, improve quality of life for large numbers of children and their families.

Acknowledgments

Acknowledgements: The Connected Bradford project is a Northern Health Science Alliance led programme funded by the Department of Health and delivered by a consortium of academic and NHS organisations across the north of England. The work uses data provided by patients and collected by the NHS as part of their care and support. The views expressed are those of the author(s) and not necessarily those of the NHSA, NHS or the Department of Health. We would like to acknowledge the support of Cathy Hulin and Poppy Konstantopoulou.

Funding: This research received no specific grant from any funding agency in the public, commercial or not-for-profit sectors.

Competing interests: The authors have declared that no competing interests exist.

Authors contributions: Contributed to the writing of the manuscript: BW, MM-W, BK, SW, DS, FM, KS, JB, JW. Statistical analysis BK. Agree with the manuscript’s results and conclusions: BW, MM-W, BK, SW, DS, FM, KS, JB, JW. All authors have read, and confirm that they meet, ICMJE criteria for authorship.

Abbreviations: ASD - Autism Spectrum Disorders; CMO – Chief Medical Officer; EYFSP – Early Years Foundation Stage Profile Score; NHS – National Health Service;

Provenance: Not commissioned; externally peer-reviewed

Data sharing statement: The Born in Bradford (BiB) dataset is available from: https://borninbradford.nhs.uk/research/how-to-access-data/

Confidential: For Review Only

References

1. NICE Local Government Briefings. Social and emotional wellbeing for children and young people. 25 September 2013.

2. Chief Medical Officer's annual report 2012: Our Children Deserve Better: Prevention Pays (published Oct 2013)

3. World Health Organization. The ICD-10 classification of mental and behavioural disorders: diagnostic criteria for research, 1992.

4. Baird, G., Simonoff, E., Pickles, A., Chandler, S., Loucas, T., Meldrum, D. and Charman, T. Prevalence of disorders of the autism spectrum in a population cohort of children in South Thames: the Special Needs and Autism Project (SNAP). *Lancet* 2006; 368(9531): 210-215.

5. Daniels AM, Mandell D. Explaining differences in age at autism spectrum disorder diagnosis: A critical review. *Autism* 2013; 18(5): 583-597.

6. Crane L, Chester JW, Goddard L, Henry LA, Hill E. Experiences of autism diagnosis: A survey of over 1000 parents in the United Kingdom. Autism 2016; 20(2): 153-162.

7. Care Quality Commission. Health Care for Disabled Children and Young People. 2012.

8. Pickles A, Le Couteur A, Leadbitter K, Salomone E, Cole-Fletcher R, Tobin H, Gammer I, Lowry J, Vamvakas G, Byford S, Aldred C. Parent-mediated social communication therapy for young children with autism (PACT): long-term follow-up of a randomised controlled trial. *The Lancet*, 2016; 388(10059): 2501-2509.

9. Daniels AM, Halladay AK, Shih A, Elder LM, Dawson G. Approaches to enhancing the early detection of autism spectrum disorders: a systematic review of the literature. *Journal of the American Academy of Child & Adolescent Psychiatry,* 2014; 53(2): 141-152.

10. Wright J, Small N, Raynor P, Tuffnell D, Bhopal R, Cameron N, Fairley L, Lawlor DA, Parslow R, Petherick ES.
and Pickett KE.. Cohort Profile: the Born in Bradford multi-ethnic family cohort study. *International Journal of Epidemiology* 2013; 42(4): 978–91.

11. Hagberg, K,W., & Jick, S.S. Validation of autism spectrum disorder diagnoses recorded in the Clinical Practice Research Datalink, 1990–2014. *Clinical Epidemiology*, 2017; *9*, p.475

12. StataCorp. Stata Statistical Software: Release 13. College Station, Texas: StataCorp LP: 2013.

13. Williams R. Using the margins command to estimate and interpret adjusted predictions and marginal effects. *Stata Journal* 2012; 12: 308–331.

Confidential: For Review Only

14. Falkmer T, Anderson K, Falkmer M, Horlin C. Diagnostic procedures in autism spectrum disorders: a systematic literature review. *European child & adolescent psychiatry* 2013; 22(6): 329-340.

15. Towle PO, Patrick PA. Autism Spectrum Disorder Screening Instruments for Very Young Children: A Systematic Review. *Autism Research and Treatment*, 2016.

16. Kelly B, Williams S, Collins S, Mushtaq F, Mon-Williams M, Wright B, Mason D, Wright J. The association between socioeconomic status and autism diagnosis in the United Kingdom for children aged 5–8 years of age: Findings from the Born in Bradford cohort. *Autism* 2017; Nov 7:1362361317733182.

17. Schneeweiss S. Learning from big health care data. *New England Journal of Medicine* 2014; 370(23): 2161-2163.

Figure legends

Figure 1: Recruitment flow chart

Figure 2: Early Years Foundation Stage Profile (EYFSP): Weighted sub-score

Figure 3: Distribution of EYFSP Scores - standardised with standard deviation shown

Figure 4: Predicted rates of ASD for children by (A) total EYFSP score and (B) five item EYFSP sub-score (designed to map more closely to items that measure factors associated with autism)

Confidential: For Review Only

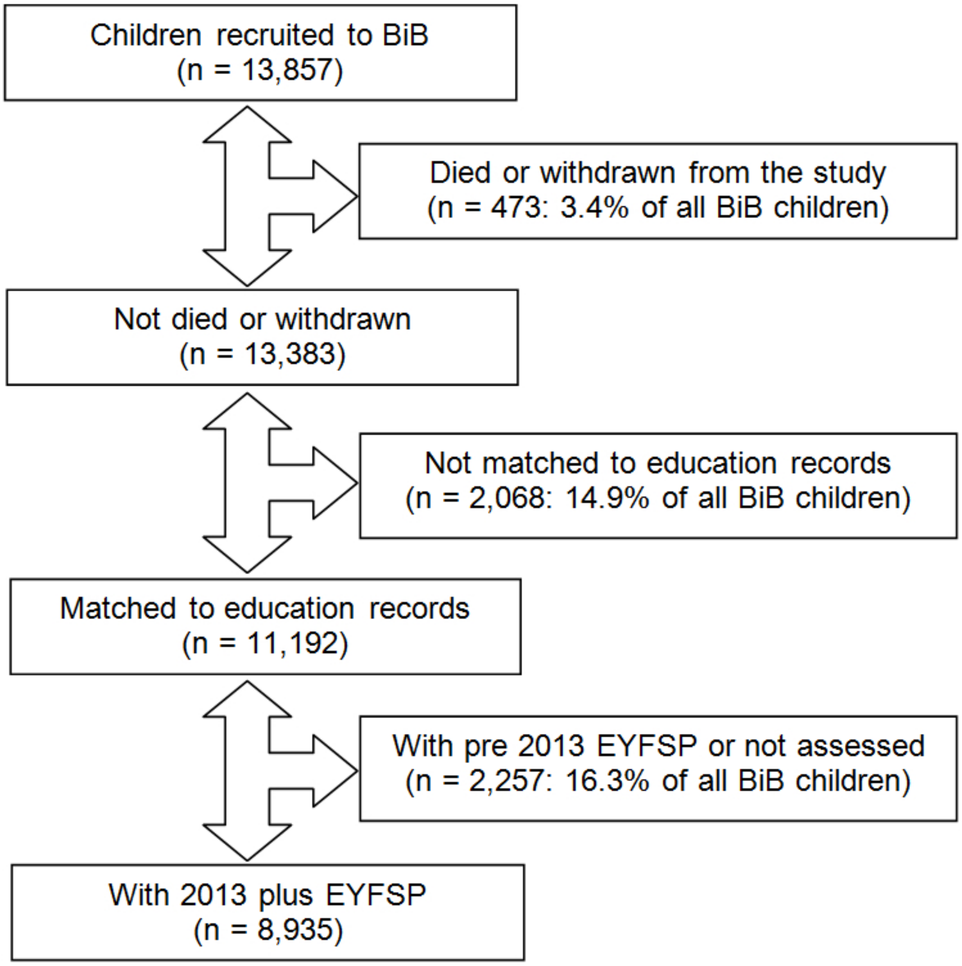

206x203mm (96 x 96 DPI)

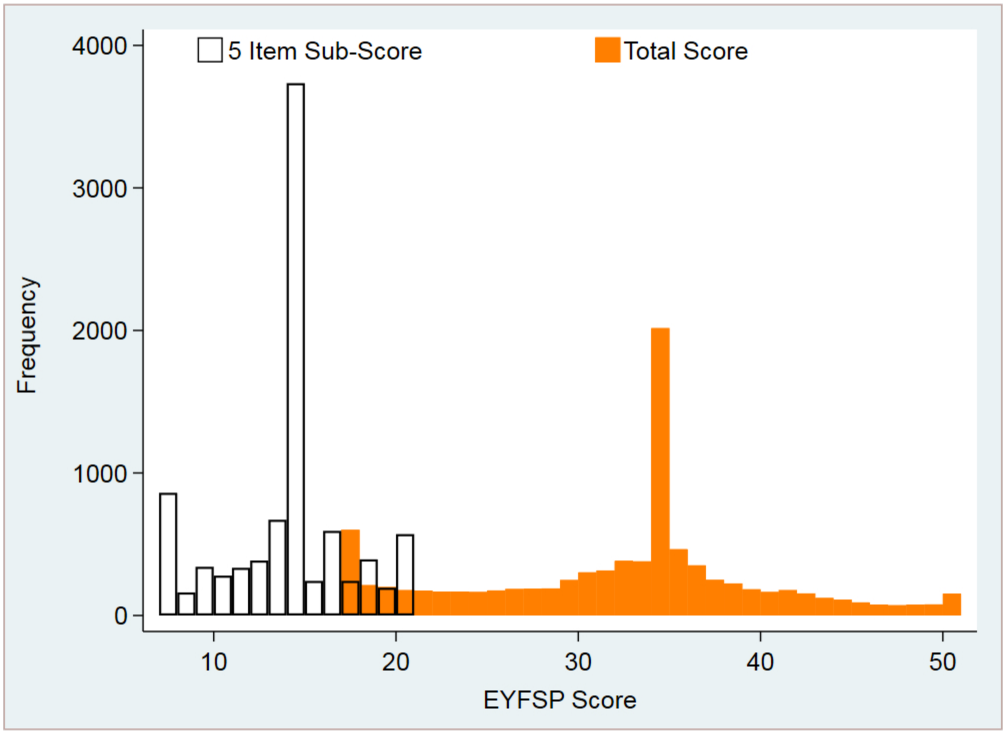

352x256mm (72 x 72 DPI)

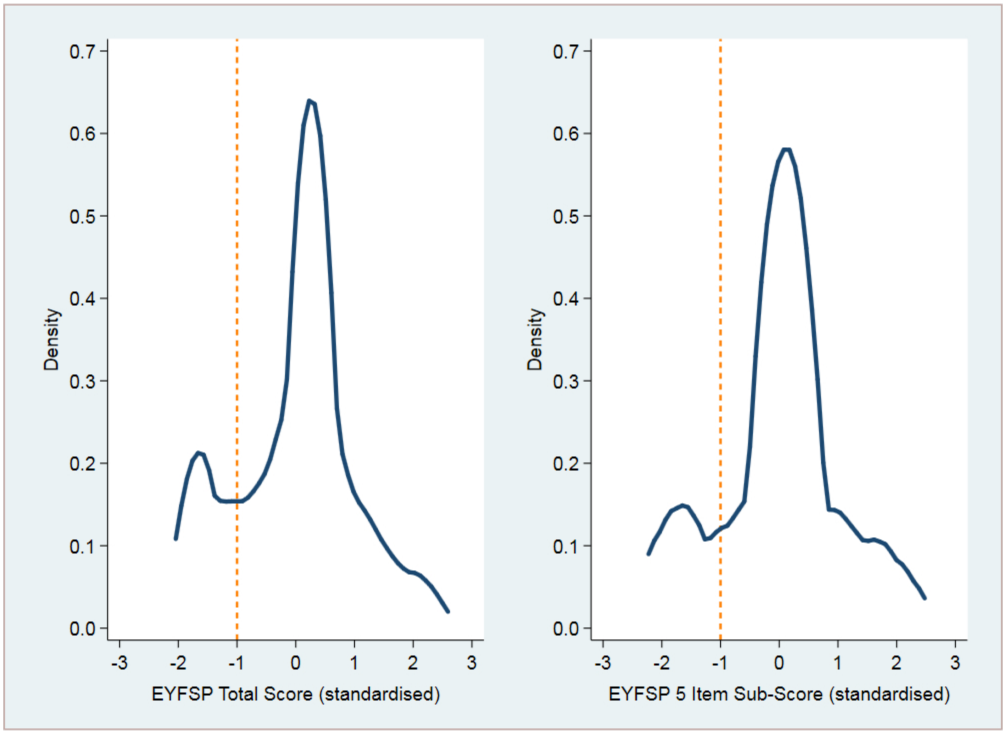

352x256mm (72 x 72 DPI)

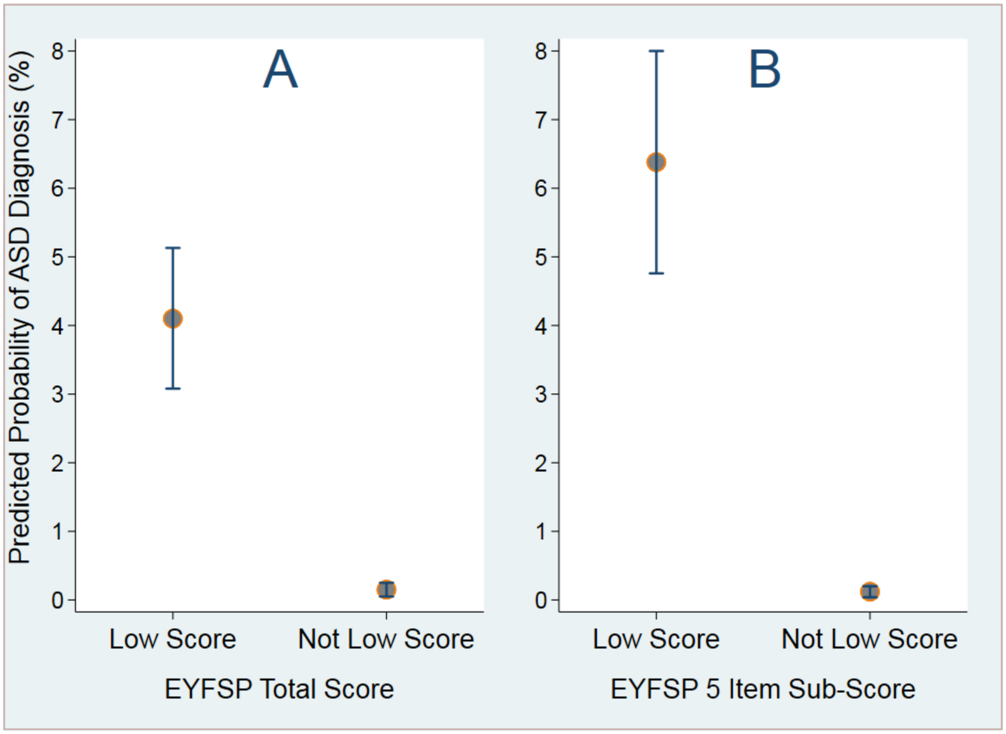

352x256mm (72 x 72 DPI)

Supplementary material 1: Results of logistic regression models

Logistic Regression Models	EYFSP Total Score			EYFSP 5 Item Sub-Score		
	Odds Ratio	95% Confidence Intervals Low	High	Odds Ratio	95% Confidence Intervals Low	High
**Univariate: EYFP score**						
Not low EYSPF score (reference)						
Low EYSFP score	23.7*	12.9	43.8	39.9*	21.6	73.8
**Multivariate: EYFP score and covariates**						
Not low EYSPF score (reference)						
Low EYSFP score	29.4*	14.4	59.8	58.1*	27.4	123.0
Gender: Female (reference)						
Gender: Male	2.87*	1.56	5.29	2.38*	1.28	4.41
Child ethnicity: White British (reference)						
Pakistani Heritage	0.45*	0.27	0.76	0.42*	0.24	0.71
Other	0.69	0.33	1.41	0.63	0.30	1.32
Receiving free school meals (reference)						
Not receiving free school meals	2.29*	1.25	4.19	2.39*	1.30	4.41
Age (years) at GP data extract: Five (reference)						
Six	1.60	0.54	4.76	1.16	0.38	3.54
Seven	0.94	0.31	2.80	0.67	0.22	2.05
Eight plus	0.93	0.31	2.82	0.71	0.23	2.19

* = significant at the 0.05 level

Confidential: For Review Only

Supplementary material 2: Read codes for autism

We used the Read codes below to identify children with a diagnosis of autism. This was supplemented by a free text search (which did not identify further cases).

**Read code**	**Read code description**
**ASDs**	
E140.00	Infantile autism
E140000	Active infantile autism
E140100	Residual infantile autism
E140.12	Autism[**]
E140.13	Childhood autism
E140000	Active infantile autism
E140z00	Infantile autism NOS
Eu84000	[X]Childhood autism
Eu84011	[X]Autistic disorder[**]
Eu84012	[X]Infantile autism
Eu84100	[X]Atypical autism
Eu84z11	[X]Autistic spectrum disorder
Eu84500	[X]Asperger's syndrome[**]

Confidential: For Review Only

Supplementary material 3: The distribution of EYFSP scores for children with and without a diagnosis of autism spectrum disorder

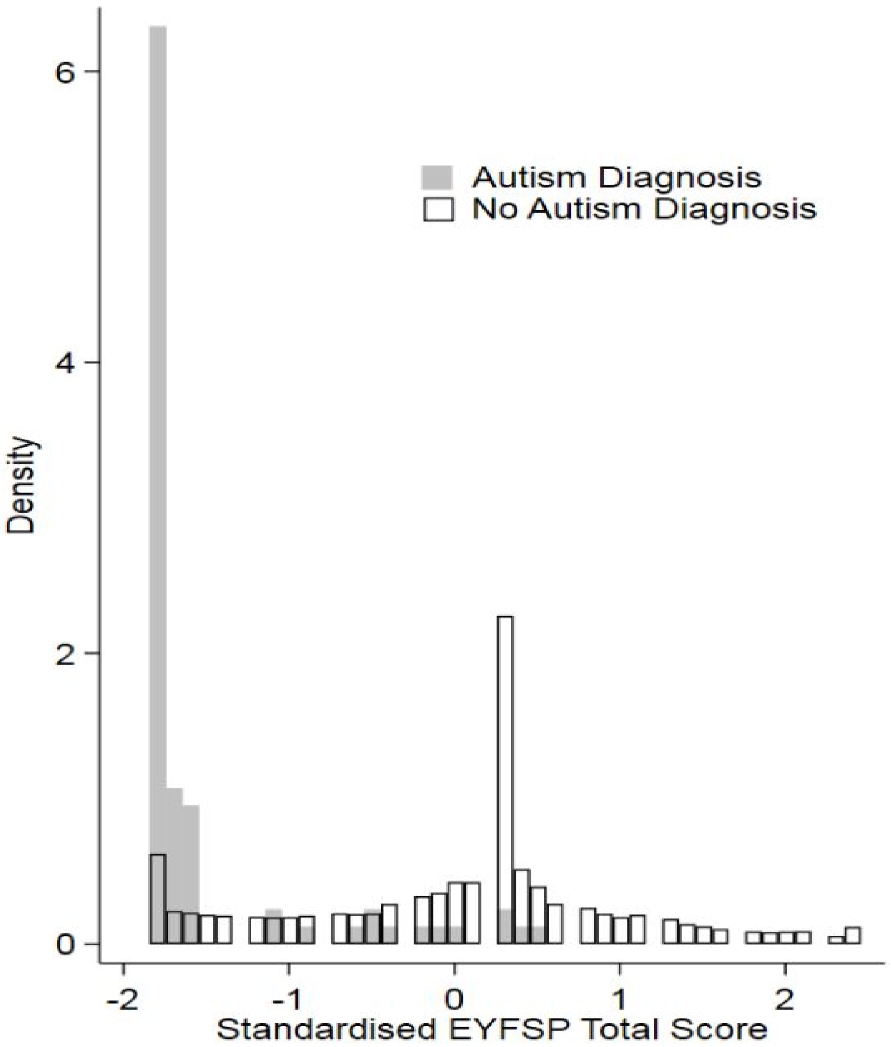

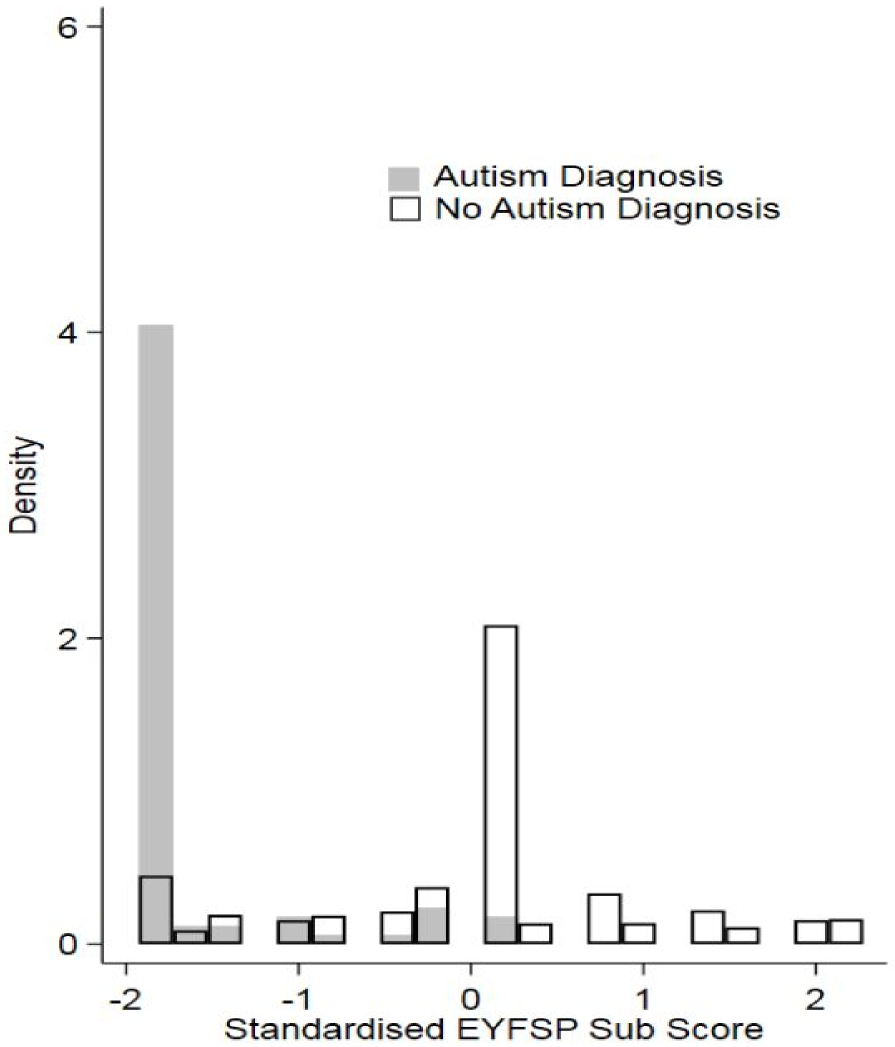

Supplement: Author's manuscript [file bmjpo-2019-000483.draft_revisions.pdf]
